# The fibronectin type-III (FNIII) domain of ATF7IP contributes to efficient transcriptional silencing mediated by the SETDB1 complex

**DOI:** 10.1186/s13072-020-00374-4

**Published:** 2020-11-30

**Authors:** Takeshi Tsusaka, Kei Fukuda, Chikako Shimura, Masaki Kato, Yoichi Shinkai

**Affiliations:** 1 Cellular Memory Laboratory, RIKEN Cluster for Pioneering Research, Wako, 351-0198 Japan; 2 Present Address: Eli and Edythe Broad Center for Regenerative Medicine and Stem Cell Research, Department of Orthopedic Surgery, University of California, San Francisco, San Francisco, CA USA; 3 Present Address: Laboratory for Transcriptome Technology, RIKEN Center for Integrative Medical Sciences (IMS), Yokohama, 230-0045 Japan

**Keywords:** ATF7IP/AM/MCAF1, SETDB1/ESET, H3K9me3, FNIII domain, Transgene silencing, ZMYM2, MGA

## Abstract

**Background:**

The histone methyltransferase SETDB1 (also known as ESET) represses genes and various types of transposable elements, such as endogenous retroviruses (ERVs) and integrated exogenous retroviruses, through a deposition of trimethylation on lysine 9 of histone H3 (H3K9me3) in mouse embryonic stem cells (mESCs). ATF7IP (also known as MCAF1 or AM), a binding partner of SETDB1, regulates the nuclear localization and enzymatic activities of SETDB1 and plays a crucial role in SETDB1-mediated transcriptional silencing. In this study, we further dissected the ATF7IP function with its truncated mutants in *Atf7ip* knockout (KO) mESCs.

**Results:**

We demonstrated that the SETDB1-interaction region within ATF7IP is essential for ATF7IP-dependent SETDB1 nuclear localization and silencing of both ERVs and integrated retroviral transgenes, whereas its C-terminal fibronectin type-III (FNIII) domain is dispensable for both these functions; rather, it has a role in efficient silencing mediated by the SETDB1 complex. Proteomic analysis identified a number of FNIII domain-interacting proteins, some of which have a consensus binding motif. We showed that one of the FNIII domain-binding proteins, ZMYM2, was involved in the efficient silencing of a transgene by ATF7IP. RNA-seq analysis of *Atf7ip* KO and WT or the FNIII domain mutant of ATF7IP-rescued *Atf7ip* KO mESCs showed that the FNIII domain mutant re-silenced most de-repressed SETDB1/ATF7IP-targeted ERVs compared to the WT. However, the silencing activity of the FNIII domain mutant was weaker than that of the ATF7IP WT, and some of the de-repressed germ cell-related genes in *Atf7ip* KO mESCs were not silenced by the FNIII domain mutant. Such germ cell-related genes are targeted and silenced by the MAX/MGA complex, and MGA was also identified as another potential binding molecule of the ATF7IP FNIII domain in the proteomic analysis. This suggests that the FNIII domain of ATF7IP acts as a binding hub of ATF7IP-interacting molecules possessing a specific interacting motif we named FAM and contributes to one layer of the SETDB1/ATF7IP complex-mediated silencing mechanisms.

**Conclusions:**

Our findings contributed to further understanding the function of ATF7IP in the SETDB1 complex, revealed the role of the FNIII domain of ATF7IP in transcriptional silencing, and suggested a potential underlying molecular mechanism for it.

## Background

Gene expression patterns play a fundamental role in a variety of biological processes. In addition to the underlying DNA sequence and its methylation status, chromatin status also influences gene expression. Multiple chemical modifications occur in histone proteins, which are the basic chromatin components, and they control multilayered chromatin structure and function. Methylation of lysine residues is a mark of both active and repressive gene expression, depending on the methylated residue, the methylation degree, and the chromatin region where the methylation happened [1]. Methylated histone H3 lysine 9 (H3K9) is associated with gene silencing, and it is dynamically regulated by several methyltransferases and demethylases. We have shown that in mESCs, one of the lysine methyltransferases, SETDB1 (also known as ESET) suppresses the expression of Class I and II endogenous retroviruses (ERVs) by depositing H3K9me3 marks [2, 3]. TRIM28 (also known as KAP1 or TIF1B) and TRIM28-associated nucleic acid-binding zinc-finger proteins (ZFPs), including KRAB-ZFPs and YY1, play an important role in SETDB1 targeting to ERVs and silencing them [4–6]. In addition, the recent studies in human cells have shown that the human silencing hub (HUSH) complex, comprised MPP8, FAM208A (also known as TASOR), and Periphilin, recruits SETDB1 to a transgene that is integrated into heterochromatin to induce H3K9me3-mediated silencing of its expression [7]. However, differences between the two silencing mechanisms have not yet been fully clarified.

ATF7IP (also known as AM or MCAF1) is a binding partner of SETDB1 [8, 9]. Loss of ATF7IP results in the de-repression of SETDB1-regulated genes, ERVs, and the transgene integrated by retroviruses, concomitant with a decreased H3K9me3, which are similar to that conferred by SETDB1 inactivation, but these phenotypes are weaker than those observed in SETDB1 inactivation [10–15]. Recently, we reported one role of ATF7IP in SETDB1, in which ATF7IP regulates SETDB1 nuclear localization and increased levels of its ubiquitinated and more enzymatically active forms [16]. However, given that SETDB1 can be imported into the nucleus and recruited to the target loci without ATF7IP [16, 17], the residual SETDB1 in the nucleus could execute a large part of its silencing function even in the absence of ATF7IP, which is consistent with the weaker phenotype of the loss of ATF7IP. Prior studies identified two functional regions within human ATF7IP: SETDB1- and MBD1-binding regions [9, 18]. Since the MBD1-binding region is also characterized as a fibronectin type-III (FNIII) domain, we referred to the region hereinafter as the “FNIII domain”. Although the residues 562–817 within human ATF7IP were shown to be essential for binding to SETDB1 [9], the functional requirement of this binding region has not been studied yet. The FNIII domain was shown to be essential for the binding of ATF7IP to MBD1, and this interaction is mediated by residues 529–592 of human MBD1, which is included in the transcriptional repression domain (TRD) of MBD1 [18]. Substitutions with arginine (R) at residues I576 and L579 of human MBD1 disrupted its interaction with human ATF7IP as well as its transcriptional repression [9, 19–21]. To further understand the function of ATF7IP in transcriptional silencing, we generated a SETDB1-binding domain and FNIII domain deletion mutant of mouse ATF7IP and performed rescue experiments of our established *Atf7ip* KO mESCs with these two deletion mutants.

## Results

### Distinct functional requirements of the SETDB1-binding region and the FNIII domain in ATF7IP-dependent retroelement silencing

**Fig. 1:**
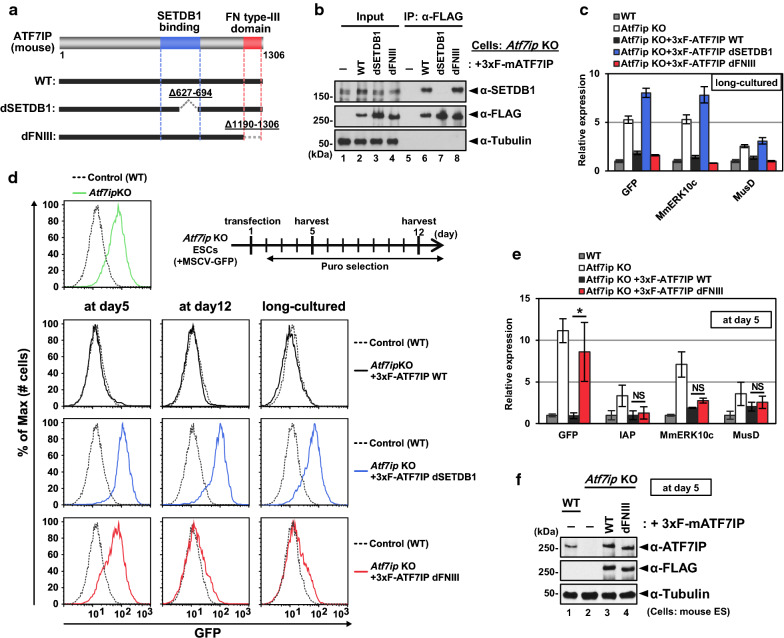
Molecular mapping analysis of ATF7IP in mESCs. **a** Domain architecture of mouse ATF7IP protein. Deletion mutants used in this study are shown. **b** Interaction of SETDB1 with each 3xFLAG-ATF7IP expressed in *Atf7ip* KO mESCs. Co-IP assay was performed with the cell lines using anti-FLAG and shows that the WT and the dFNIII mutant, but not the dSETDB1 mutant, bound to SETDB1. **c** RT-qPCR analysis was performed with long-cultured cells after the transfection. RNA expression was normalized to *Hprt* expression and is shown relative to the level in WT cells. Data are mean ± SD; *n* = 3, technical replicates. **d** MSCV-GFP expression was analyzed by flow cytometric analysis at indicated time points. The transfected cells were subjected to continuous drug selection from 1 day after the transfection. **e** RT-qPCR analysis was performed with samples collected at day 5. RNA expression was normalized to *Hprt* expression and is shown relative to the level in WT cells. Data are mean ± SEM; *n* = 4 without IAP (*n* = 3) from four or three experiments. NS: *P* > 0.05, *P < 0.05 by unpaired Student’s t-test. **f** WB analysis was performed with samples collected at day 5. Comparative expression between the WT and the dFNIII mutant ATF7IP was confirmed

We have previously established *Atf7ip* KO cells using mESCs infected with the murine stem cell virus (MSCV) carrying the GFP gene as a background [22] and observed that the *Atf7ip* KO ESCs showed increased expression of SETDB1-regulated ERVs and the MSCV-GFP reporter [16]. For rescue experiments with ATF7IP, we used a piggyBac transposase-based vector for the expression of 3xFLAG-tagged mouse ATF7IP with either WT or each domain’s deletion mutants: dSETDB1 lacking residues 627–694, which is within the corresponding SETDB1 binding domain of mouse ATF7IP and covering two estimated α-helix regions, and dFNIII lacking residues 1190–1306 of the FNIII domain, which is highly conserved between human and mouse ATF7IP (Fig. 1a and Additional file 1: Fig. S1). These piggyBac transposase-based vectors can be integrated into the host genome and stably express exogenous FLAG-tagged ATF7IPs. By the transfection of each plasmid and continuous drug selection, we established *Atf7ip* KO mESCs expressing 3xFLAG-ATF7IP-WT, dSETDB1, or dFNIII mutants and performed a co-immunoprecipitation (co-IP) assay with these cell lines using a FLAG M2 affinity gel. As expected, the dSETDB1 mutant could not co-IP endogenous SETDB1, while the WT and the dFNIII mutant could (Fig. 1b). We then examined the RNA expression of exogenous MSCV-GFP and ERVs regulated by ATF7IP and SETDB1 under long-term culturing conditions (greater than 2 weeks). As shown in Fig. 1c, the ATF7IP WT and the dFNIII mutant re-silenced the de-repressed MSCV-GFP and ERV expression, while the dSETDB1 mutant did not, suggesting that its interaction with SETDB1 is essential for the role of ATF7IP in transcriptional silencing.

We next examined the silencing kinetics of these rescued cells by taking advantage of the MSCV-GFP reporter (Fig. 1d). We transfected 3xFLAG-ATF7IP with either WT, dSETDB1, or dFNIII expression vectors into *Atf7ip* KO mESCs, and the transfected cells were selected by continuous puromycin treatment. At day 5 after transfection, GFP expression levels were analyzed by flow cytometry. As expected, the WT-rescued cells showed low GFP expression, similar to that of the parental *Atf7ip* WT cells, and the dSETDB1-rescued cells showed higher GFP expression, as seen in the *Atf7ip* KO cells. Unexpectedly, the expression of the dFNIII mutant could not re-silence MSCV-GFP expression on day 5. However, the higher GFP expression in the dFNIII-expressing cells was repressed to the WT level after culturing them for an additional seven days (at day 12). Consistent with this observation, the dFNIII-rescued cells at day 5 showed higher expression of GFP mRNA as compared to that of the WT-rescued cells (Fig. 1e). We then confirmed the expression of exogenous ATF7IP protein between the WT- and dFNIII-rescued cells at day 5 by western blot analysis (Fig. 1f). Interestingly, ERVs were re-silenced by the expression of the dFNIII mutant, similar to WT expression, even at day 5 (Fig. 1e). These results suggest that the FNIII domain plays a role in ATF7IP-mediated transcriptional silencing under certain condition.

### Interaction with SETDB1, but not the FNIII domain of ATF7IP, is required for ATF7IP-dependent SETDB1 nuclear localization

**Fig. 2:**
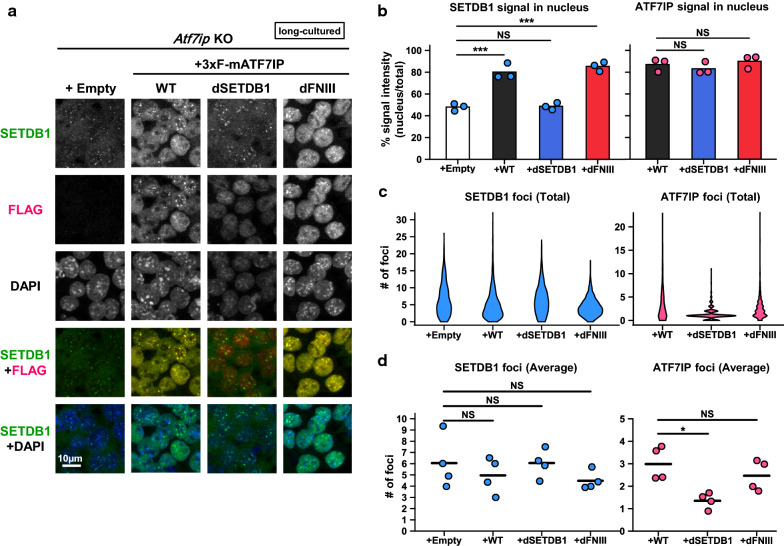
The SETDB1-binding region, but not the FNIII domain, within ATF7IP is required for normal localization of SETDB1. **a** IF analysis shows that exogenous expression of 3xFLAG-ATF7IP WT and dFNIII mutant, but not dSETDB1 mutant, rescues the abnormal localization of SETDB1 in *Atf7ip* KO mESCs. Representative images are shown, and the quantitative analyses are shown in F–H. Scale bar: 10 µm. **b** SETDB1 (left) and 3xFLAG-ATF7IP (right) signals in the nucleus that was determined by DAPI staining were calculated. The mean from three independent experiments is shown as a bar graph with jittered points indicating the average % intensity of each experiment. Over 100 cells were analyzed per sample per experiment. NS (not significant): *P* > 0.05, ***P < 0.001 versus “ + Empty” group by Dunnett’s test. **c**, **d** SETDB1 (left) and 3xFLAG-ATF7IP (right) foci in the nucleus that was determined by DAPI staining were calculated. Violin plot for their foci numbers from all the cells analyzed are shown (**c**). The mean from four independent experiments is shown as a bar with jittered points indicating the average number of each experiment (**d**)

We have recently reported that ATF7IP regulates SETDB1’s nuclear localization by antagonizing and enhancing its nuclear export and import, respectively [16]. Therefore, we determined whether the expression of the dSETDB1 or FNIII mutant can rescue the cytoplasmic accumulation phenotype of SETDB1 in *Atf7ip* KO mESCs. We examined SETDB1 localization in the long-cultured 3xFLAG-tagged ATF7IP WT-, dSETDB1-, and dFNIII-rescued cells by immunofluorescence (IF) analysis. The WT and the two mutants of 3xFLAG-tagged ATF7IP were all localized in the nucleus (Fig. 2a; quantification in Fig. 2b, right). The expression of the ATF7IP WT and the dFNIII mutant in *Atf7ip* KO cells restored the SETDB1’s nuclear localization (Fig. 2a; quantification in Fig. 2b, left) without significant changes in nuclear foci numbers (Fig. 2c, d, left). Both the ATF7IP WT and the dFNIII mutant also co-localized with SETDB1 in the nucleus and in the nuclear foci (Fig. 2a). In contrast, the dSETDB1 mutant could not rescue the cytoplasmic localization of SETDB1 (Fig. 2a; quantification in Fig. 2b, left), and the number of dSETDB1 nuclear foci was reduced (Fig. 2a; quantification in Fig. 2c, d, right). The dFNIII-rescued cells also showed that SETDB1 and exogenous ATF7IP were localized in the nucleus as efficiently as those in the ATF7IP WT-rescued *Atf7ip* KO cells five days after transfection (Additional file 2: Fig. S2A–C). These results suggest that the regulation of SETDB1 nuclear localization by ATF7IP requires their interaction and that a delayed silencing of MSCV-GFP in dFNIII mutant-rescued cells does not seem to be caused by SETDB1’s delayed nuclear localization or mislocalization.

### Identification of ATF7IP FNIII domain-binding proteins

**Fig. 3:**
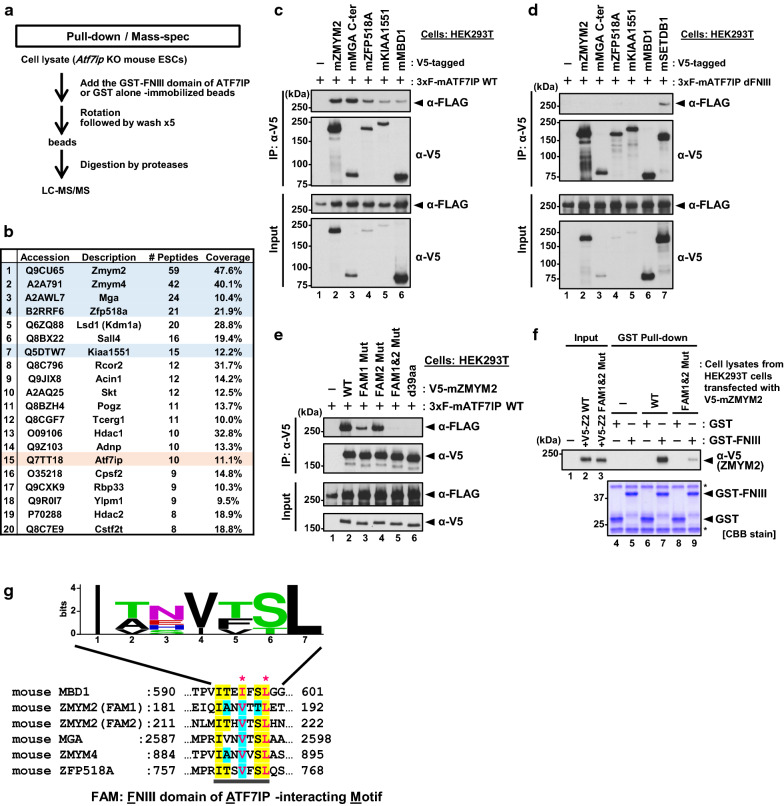
Identification of binding proteins for ATF7IP’s C-terminal FNIII domain. **a** An experimental design for identification of binding proteins of ATF7IP’s FNIII domain by pull-down assay followed by LC–MS/MS analysis. **b** A list of top 20 proteins identified in the proteomic analysis. The proteins which was confirmed to interact with ATF7IP by independent experiment are filled in blue. ATF7IP is filled in red. **c** Co-IP assay performed with HEK293T cells transiently co-transfected with 3xFLAG-tagged ATF7IP WT and V5-tagged proteins. MBD1, a known ATF7IP interactor, was used as a positive control. All the high-ranked proteins bound to ATF7IP. **d** Co-IP assay performed with HEK29T cells transiently co-transfected with 3xFLAG-tagged ATF7IP dFNIII mutant and V5-tagged indicated proteins plus SETDB1. The FNIII mutant bound to SETDB1, but not to other proteins. **e** Co-IP assay performed with HEK29Tcells transiently co-transfected with 3xFLAG-tagged ATF7IP WT and V5-tagged ZMYM2 WT or mutants. FAM1 Mut possesses substitutions, V187R and L190R; FAM2 Mut possesses substitutions V217R and L220R; FAM1&FAM2 Mut possesses all the four substitutions; Δ39aa mutant lacks the region of 182–220 amino acids of ZMYM2. **f** FNIII domain of ATF7IP is sufficient for the interaction with ZMYM2. GST-pull down assay with recombinant FNIII domain of ATF7IP and cells lysates from HEK293T cells transiently transfected with V5-ZMYM2 WT or FAM1&2 Mut was performed. The pulled-down samples were analyzed by WB. **g** Alignment of MBD1-resembled sequences within the indicated proteins and their sequence logo. Asterisk indicates important residues for the interaction with ATF7IP. The substitutions of the I/V and L with R disrupted the interaction with ATF7IP. This consensus sequence is proposed as a FN III domain of ATF7IP-interacting Motif (FAM). The sequence logo was created by WEBLOGO (version 2.8.2)

We further sought to reveal the underlying mechanism for the inefficient re-silencing of the MSCV-GFP reporter transgene by the dFNIII mutant (Fig. 1d). Since the FNIII domain functions as a binding domain for MBD1 in human cell lines [9, 18], we searched for binding proteins for the FNIII domain of ATF7IP in mESCs. For this, we performed a proteomic analysis with the recombinant FNIII domain of mouse ATF7IP and nuclear lysates of mESCs (Fig. 3a). The nuclear fractions from *Atf7ip* KO mESCs were incubated with a GST-tagged FNIII domain, produced in, and purified from *E. coli*. After purification with glutathione beads, the bound proteins with the FNIII domain were identified by liquid chromatography followed by tandem mass spectrometry (LC–MS/MS) analysis (Fig. 3b, the full list is in Additional file 3: Table S1). We identified over 20 proteins enriched in the FNIII domain-pulled-down sample, with a high coverage, and found some known protein networks, including a ZMYM2 (also known as ZNF198)-LSD1 (also known as KDM1A)-HDAC complex [23, 24], by STRING analysis (Additional file 4: Fig. S3). Unexpectedly, we could not recover MBD1 in our proteomic analysis, suggesting that MBD1 may not exist in *Atf7ip* KO mESCs or that their interaction may not occur significantly in mESCs. Using a co-IP experiment with transient ectopic expression in HEK293T cells, we validated the interaction of full-length ATF7IP with several high-ranked candidates, including ZMYM2, MGA (residues 2362–3003), ZFP518A, and KIAA1551, and a known interactor MBD1 (Fig. 3c). As expected, the dFNIII mutant could not bind to these proteins, but it could bind to SETDB1 (Fig. 3d), supporting the hypothesis that these newly identified interactors, as well as MBD1, can bind to the FNIII domain of ATF7IP.

To examine which region of ZMYM2, which is a top-ranked protein in our proteomic analysis, is essential for its interaction with ATF7IP, we performed co-IP experiments with a series of truncated mutants of ZMYM2 using HEK293T cells (Additional file 5: Fig. S4A–D). These results suggest that residues 181–350 within ZMYM2 seem to be important for its interaction with ATF7IP. Visual inspection of this region revealed that it contains two sequences (referred to as FNIII domain of ATF7IP-interacting motif 1 (FAM1) and FAM2, explained later) similar to an “ITEFSL” sequence within the TRD of MBD1, which was shown to be essential for its binding to the FNIII domain [18]. As substitutions of isoleucine (I) and leucine (L) to arginine (R) within this sequence perturbed the interaction between MBD1 and the FNIII domain [9, 18], we wondered whether similar mutations at the corresponding “V” and “L” residues of FAM1 and/or FAM2 within ZMYM2 can affect the interaction between ZMYM2 and ATF7IP (see Additional file 6: Fig. S5A). We transfected 3xFLAG-ATF7IP with either the control empty vector, V5-ZMYM2-WT, FAM1 mutant (V187R/L190R), FAM2 mutant (V217R/L220R), FAM1 and FAM2 double mutant, or a d39aa mutant that lacks residues 182–220 into HEK293T cells and performed co-IP experiments with an anti-FLAG M2 affinity gel (Fig. 3e). The results showed that the mutations at either FAM1 or FAM2 impaired the interaction, and that the FAM1 and 2 mutant or the d39aa mutant completely failed to co-IP ATF7IP, suggesting that both FAM1 and FAM2 contribute to the binding with ATF7IP. We further examined the interaction of ZMYM2 with ATF7IP using a GST pull-down assay (Fig. 3f). The HEK293Tcell lysates transfected with V5-ZMYM2 WT or FAM1&2 Mut were pulled-down with a recombinant GST or a recombinant GST-FNIII domain. Western blot analysis showed that the GST-FNIII domain, but not GST alone, bound to ZMYM2 WT, and that the binding of the FAM1 and 2 mutant to ATF7IP was severely impaired compared to that of the ZMYM2 WT. These data suggest that ZMYM2 binds to the FNIII domain of ATF7IP via its FAM1 and FAM2 motifs. We then examined primary sequences of the identified FNIII-binding proteins and found that MGA, ZMYM4, and ZFP518A possess an “ITEFSL”-like sequence (Fig. 3g). We showed that mutations on the motif abolished the interaction of those proteins with ATF7IP (Additional file 6: Fig. S5A–C). Therefore, we proposed that the “ITEFSL”-like sequences are a consensus binding motif for the FNIII domain of ATF7IP, and referred to as FAM.

### ZMYM2 is involved in the efficient silencing of exogenous provirus reporter by ATF7IP

**Fig. 4:**
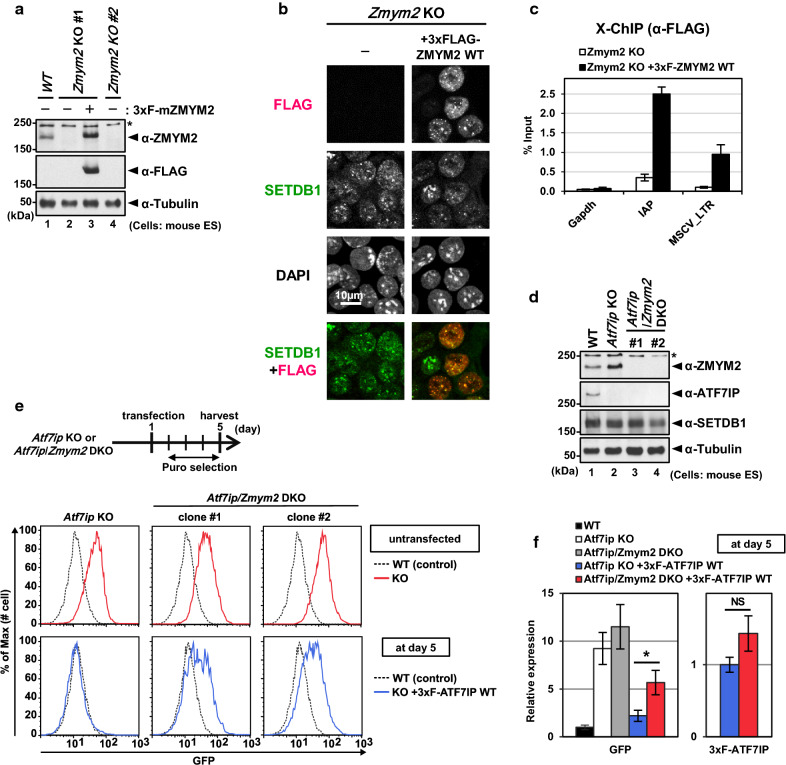
ZMYM2 partly mediates efficient re-silencing of MSCV-GFP reporter by ATF7IP. **a** WB analysis confirms no expression of ZMYM2 in *Zmym2* KO cell lines and shows an exogenous expression of 3xFLAG-ZMYM2. **b** IF analysis shows the co-localization of 3xFLAG-ZMYM2 with SETDB1’s nuclear foci. **c** X-ChIP analysis with anti-FLAG antibody at the indicated genomic loci in *Zmym2* KO mESCs and the *Zmym2* KO cells rescued by 3xFLAG-ZMYM2. *Gapdh* gene was used as a negative control. The ChIP enrichment levels are shown as mean ± SEM; *n* = 3 from three experiments. **d** WB analysis confirms no expression of both ATF7IP and ZMYM2 in *Atf7ip*/*Zmym2* DKO cells. **e** MSCV-GFP expression was analyzed by flow cytometric analysis at day 5 after the transfection. *Atf7ip*/*Zmym2* DKO cells show higher expression of GFP, compared to the parental WT cells. **f** RT-qPCR analysis was performed with samples collected at day 5 after the transfection. RNA expression was normalized to *Hprt* expression and is shown relative to the level in WT cells (left) or *Atf7ip* KO cells transfected with 3xFLAG-ATF7IP WT. Data are mean ± SEM; *n* = 4 from four experiments. NS: *P* > 0.05, *P < 0.05 by unpaired Student’s *t* test

Among the identified binding proteins for the FNIII domain of ATF7IP, we focused our attention on the top-ranked protein ZMYM2, which has two FAMs, and may function with the LSD-HDAC1 repressor as a complex [23, 24]. We established *Zmym2* KO mESCs using CRISPR/Cas9 technology. mESCs harboring the hCas9 and MSCV-GFP reporter were transfected with an expression vector for gRNA targeting the mouse *Zmym2* gene. We observed a slight increase in the MSCV-GFP reporter in the *Zmym2*-gRNA-transfected cells by flow cytometry analysis (Additional file 7: Fig. S6A) and sorted the cell populations with high GFP intensity. The sorted cells were cloned, and the ZMYM2 expression in the cloned cell lines were subsequently analyzed. We finally isolated two independent clones of *Zmym2* KO mESCs, as evidenced by western blot using an anti-ZMYM2 antibody (Fig. 4a). We found that both established *Zmym2* KO cell lines showed GFP expression equivalent to the parental WT cells (Additional file 7: Fig. S6B), resembling the case of the ATF7IP FNIII-rescued *Atf7ip* KO mESCs after long-term culture (Fig. 1d). We then transfected *Zmym2* KO cells with a 3xFLAG-tagged ZMYM2 WT expression vector and confirmed their expression (Fig. 4a). By using the 3xFLAG-tagged ZMYM2-rescued cells, we observed the co-localization of 3xFLAG-ZMYM2 with endogenous SETDB1 at the foci in the nucleus by IF analysis (Fig. 4b), suggesting a potential function of ZMYM2 in interacting with SETDB1 and ATF7IP. Furthermore, we found an enrichment of the FLAG-tagged ZMYM2 at the SETDB1/ATF7IP-target genomic regions, including the LTR of MSCV-GFP (Fig. 4c).

To examine the potential involvement of ZMYM2 in the re-silencing of the MSCV-GFP reporter by ATF7IP, we further inactivated *Zmym2* in *Atf7ip* KO mESCs. We confirmed the depletion of ZMYM2 protein in the two *Zmym2*/*Atf7ip* DKO cell lines by western blot analysis (Fig. 4d). Furthermore, we found an upregulation of ZMYM2 protein in *Atf7ip* KO mESCs as compared to the parental WT cells (Fig. 4d), suggesting the existence of a potential negative feedback mechanism. We then transfected either *Atf7ip* KO cells or *Zmym2*/*Atf7ip* DKO cell lines with 3xFLAG-ATF7IP WT and analyzed them by flow cytometric analysis five days after transfection. We found that the re-expression of ATF7IP WT in the *Zmym2*/*Atf7ip* DKO cell lines incompletely silenced the expression of MSCV-GFP reporter (Fig. 4e). Consistent with this, we observed, by RT-qPCR analysis that although the expression levels of 3xFLAG-ATF7IP WT mRNA were similar, the 3xFLAG-ATF7IP WT-rescued *Atf7ip*/*Zmym2* DKO cells showed ~ threefold increase in the expression of GFP mRNA as compared to the 3xFLAG-ATF7IP WT-rescued *Atf7ip* KO cells at day 5 after transfection (Fig. 4f). Taken together, these results suggest that ZMYM2 partly mediates the efficient re-silencing of the MSCV-GFP reporter by ATF7IP in mESCs.

### The FNIII domain of ATF7IP contributes to the efficient silencing of SETDB1 target ERVs and some MGA/MAX-targeted germ cell-related genes

**Fig. 5:**
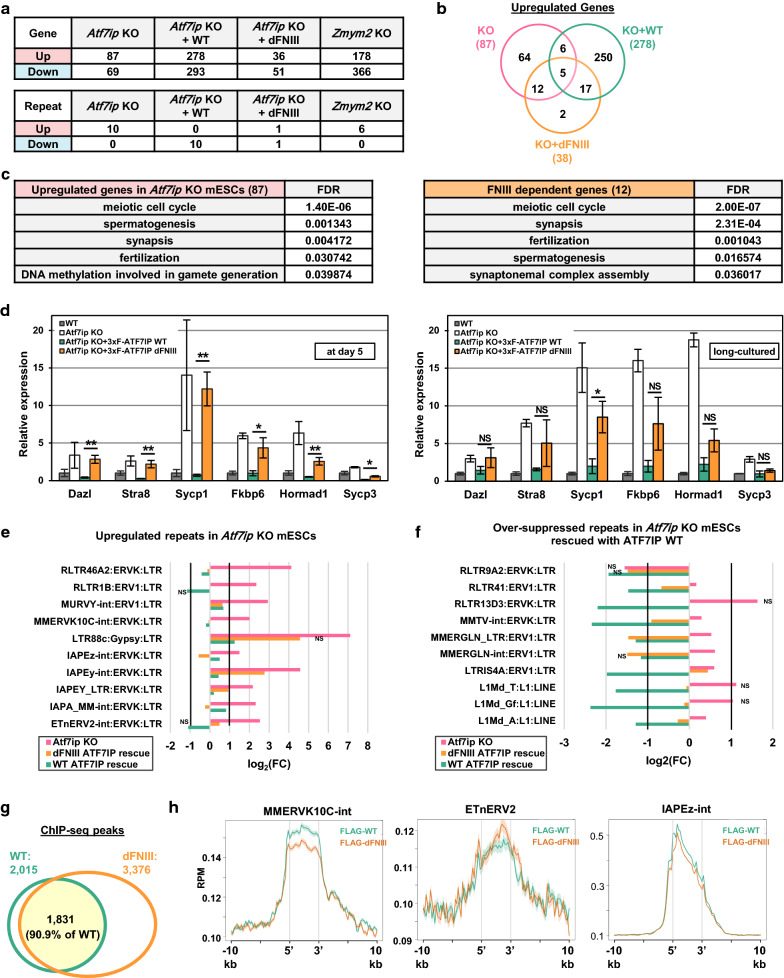
FNIII domain of ATF7IP contributes to efficient silencing of SETDB1 target ERVs and some of MGA/MAX target germ cell-related genes. **a** The number of DE genes and repeats (FDR < 0.05, FC ≥ 2) in *Atf7ip* KO, *Atf7ip* KO rescued with WT or the FNIII domain mutant of ATF7IP and *Zmym2* KO ESCs. **b** Overlap of differentially upregulated genes in *Atf7ip* KO EScs, *Atf7ip* KO ESCs rescued with WT or the FNIII domain mutant of *Atf7ip.* **c** GO term enrichment analysis for cellular component of up-regulated genes in *Atf7ip* KO ESCs (upper panel) or commonly upregulated genes in *Atf7ip* KO and *Atf7ip* KO ESCs rescued with the FNIII domain mutant of ATF7IP (lower panel). The analysis was performed by DAVID. **d** RT-qPCR analysis of germ-cell related genes in WT, *Atf7ip* KO, *Atf7ip* KO ESCs rescued with WT or the FNIII domain mutant of ATF7IP. RNA samples were collected at day 5 (left) or more than 2 weeks (right) after the transfection. RNA expression was normalized to *Hprt* expression and is shown relative to the level in WT cells. Data are mean ± SEM; *n* = 4. NS: *P* > 0.05, *P < 0.05 by unpaired Student’s *t* test. **e** Up-regulated repeats in *Atf7ip* KO mESCs. **f** Down-regulated repeats in *Atf7ip* KO mESCs expressing Atf7ip WT transgene. **g** Overlap of 3xFLAG-ATF7IP WT and FNIII domain mutant stringent peaks. **h** Enrichment of FLAG-ATF7IP in retroelements

To further elucidate the role of the FNIII domain in ATF7IP-mediated transcriptional regulation, we performed RNA-seq analysis of WT, *Atf7ip* KO, and *Atf7ip* KO stably rescued with WT or FNIII domain mutant of ATF7IP and *Zmym2* KO mESCs (Additional file 8: Fig. S7). In comparison with parental WT ESCs, 87 and 69 genes were commonly up- and downregulated, respectively, (FDR < 0.05, FC ≥ 2) in two independent *Atf7ip* KO mESC clones, TT#2-5 and TT#2-12 [16] (Fig. 5a and Additional file 9: Table. S2). A majority of the upregulated genes (76/87) in *Atf7ip* KO cells were repressed by exogenous ATF7IP WT expression (Fig. 5b). Complementation with ATF7IP WT induced a greater number of up- or downregulated genes compared to the *Atf7IP* KO (Fig. 5a). When the FNIII domain mutant was introduced into *Atf7ip* KO ESCs, a majority of the upregulated genes (70/87) in *Atf7ip* KO mESCs were reversed to WT levels (Fig. 5b). Interestingly, Gene Ontology (GO) term enrichment analysis using DAVID 6.7 [25] showed that GO terms related to the meiotic cell cycle or spermatogenesis were enriched in the 12 genes that were re-silenced in the WT rescued cells, but not in the dFNIII mutant-rescued cells (Fig. 5c). These include *Rec114, Tex11, Tex15, Fkbp6, Sycp1, Stra8,* and *Mael* (Additional file 9: Table S2). Such germ cell-related genes were also de-repressed in *Setdb1* KO mESCs [3]. Furthermore, it has been reported that these genes were also induced in the *Max* knockdown (KD) mESCs, and that some of them were de-repressed in *Atf7ip* KD mESCs [26]. RT-qPCR analysis confirmed that the de-repressed germ cell-related genes were not repressed by the FNIII domain mutant in *Atf7ip* KO ESCs at day 5 after transfection or were only partially silenced over longer culture conditions, whereas these genes were efficiently silenced by ATF7IP WT even at day 5 post-transfection (Fig. 5d). Thus, the FNIII domain has an indispensable role in the ATF7IP-mediated silencing of some MAX-regulated germ cell-related genes.

In the case of the retroelements, 10 different classes of repeats were upregulated in *Atf7ip* KO mESCs, which was consistent with previous findings [15], and all of them were repressed by exogenous ATF7IP WT expression (Fig. 5a, e). The introduction of the FNIII domain mutant also repressed most of the de-repressed repeats (9/10) (Fig. 5a, e). Interestingly, the other 10 different classes of retroelements were further downregulated in *Atf7ip* KO mESCs rescued with ATF7IP WT (Fig. 5a, f). The additionally downregulated retroelements were also SETDB1-targeted and de-repressed in *Setdb1* KO mESCs [3, 15]. Since most of the additionally downregulated repeats by exogenous ATF7IP WT expression were indeed weakly (< twofold) de-repressed in *Atf7ip* KO ESCs (Fig. 5f), SETDB1-mediated retroelement silencing might be enhanced by the overproduction of ATF7IP WT in mESCs. However, the majority of additionally downregulated repeats by exogenous ATF7IP WT expression were more mildly repressed by the FNIII domain mutant in comparison with ATF7IP WT (Fig. 5f), even though the two molecules were similarly expressed (Fig. 1b), supporting the notion that the FNIII domain of ATF7IP contributes to efficient transcriptional silencing mediated by the SETDB1 complex.

In *Zmym2* KO mESCs, multiple genes were also upregulated and downregulated (Fig. 5a). More than half of the upregulated genes (52/87) in *Atf7ip* KO mESCs were also upregulated in *Zmym2* KO mESCs, whereas a smaller portion of upregulated genes (52/178) in *Zmym2* KO mESCs were also upregulated in *Atf7ip* KO mESCs (Additional file 9: Table S2 and Additional file 10: Fig. S8A, B), suggesting that the regulation of the majority of the upregulated genes in *Zmym2* KO mESCs is independent of FNIII domain interaction. More importantly, however, among the 12 upregulated genes in *Atf7ip* KO mESCs that were not silenced by the FNIII domain mutant, 11 genes were also upregulated by ZMYM2 depletion (Additional file 9: Table. S2). We confirmed that the genes commonly upregulated in *Atf7ip* KO mESCs expressing the dFNIII mutant and the *Zmym2* KO mESCs are repressed in the *Zmym2* KO mESC complemented with 3xFLAG-ZMYM2 (Additional file 7: Fig. S6C). RNA-seq analysis showed that six different classes of repeats, which were mainly L1 elements, were upregulated in *Zmym2* KO mESCs (Fig. 5a and Additional file 10: Fig. S8C). Furthermore, the IAPEy-int retroelement, which was upregulated in *Atf7ip* KO mESCs and was not silenced by the FNIII domain mutant, was also de-repressed in *Zmym2* KO mESCs (Additional file 10: Fig. S8C and D). Thus, ZMYM2 contributes to ATF7IP FNIII domain-dependent transcriptional silencing, including MAX-targeted germ cell-related gene regulation.

Finally, we performed ChIP-seq analysis of *Atf7ip* KO mESCs rescued with 3xFLAG-tagged WT or the FNIII domain mutant of ATF7IP with an anti-FLAG antibody. We analyzed two samples for each cell type, and commonly detected peaks between two samples were defined as stringent peaks and utilized for subsequent informatics analysis. As shown in Fig. 5g, more than 90% of 3xFLAG-ATF7IP WT stringent peaks (1831/2015) overlapped with 3xFLAG-dFNIII mutant peaks. Because the number of 3xFLAG-ATF7IP WT peaks for one ChIP-seq sample was about 1/3 that of the other sample (2229 vs. 6331), the stringent peaks of 3xFLAG-ATF7IP WT might be underrepresented. When the total peaks of 3xFLAG-ATF7IP WT from two ChIP-seq samples were used for the same comparison analysis, 94.1% of the 3xFLAG-dFNIII mutant stringent peaks (3177/3376) overlapped with the 3xFLAG-WT peaks (Additional file 11: Fig. S9A). 3xFLAG-ATF7IP WP was enriched on the transcription start site of some of the upregulated germ cell-related genes in *Atf7ip* KO mESCs, but the enrichment of 3xFLAG-dFNIII mutant was lost or diminished on them (Additional file 11: Fig. S9B). We further examined 3xFLAG-ATF7IP accumulation on the retroelements that were major targets of the SETDB1/ATF7IP complex and de-repressed in *Atf7ip* or *Setdb1* KO mESCs. As shown in Fig. 5h, 3xFLAG-ATF7IP WT was enriched in the retroelements and the binding profiles of the 3xFLAG-dFNIII mutant were mostly maintained. These data indicate that the deletion of the FNIII domain does not have a strong impact on ATF7IP targeting and accumulation, especially on SETDB1 target retroelements.

## Discussion

Previous studies have revealed the importance of ATF7IP in transcriptional silencing [11–14]. Considering that ATF7IP is a relatively large protein and contains at least two functional binding surfaces for other proteins (SETDB1 and MBD1), it would be worthwhile to determine the functional requirements of each domain for transcriptional silencing by ATF7IP. We used deletion mutants of ATF7IP and provided evidence for the distinct functional requirements of the two regions of ATF7IP. In our previous study, we showed that ATF7IP plays a pivotal role in the nuclear localization of SETDB1 [16]. Our new study using the dSETDB1 mutant of ATF7IP further indicates that interaction with ATF7IP is essential for ATF7IP-dependent SETDB1 nuclear localization. On the other hand, the dFNIII mutant mostly silenced SETDB1/ATF7IP-targeted retroelements, but failed to repress some germ cell-related genes (Figs. 1c and 5c, d). To find a clue for the function of the FNIII domain, we performed a proteomic analysis for FNIII-binding proteins and identified over 20 candidate proteins, some of which were validated by independent experiments and possessed a consensus sequence, named FAM (Fig. 3). These FNIII-binding proteins may be relevant to ATF7IP functions. Among them, ZMYM2, is a member of the MYM-type zinc finger proteins and associates with a transcriptional repressor complex that contains LSD1, CoREST, and HDAC1/2 (LCH complex) via its MYM domain [23, 24]. Although the functions of ZMYM2 in transcriptional regulation have not yet been revealed, it was suggested to contribute to maintain the intact LCH complex on chromatin [24]. Here, we show that ZMYM2 partly mediates transcriptional silencing of the MSCV-GFP reporter integrated into mESCs upon the re-expression of ATF7IP (Fig. 4). ZMYM2 may recruit the LCH complex to the target loci of ATF7IP and SETDB1 to facilitate the initiation of transcriptional silencing. In addition to ZMYM2, we identified ZMYM4 as an FNIII domain of an ATF7IP-binding protein among other ZMYM family proteins in our proteomic analysis (Additional file 3: Table S1). These proteins may play a role in transcriptional silencing by ATF7IP, as ZMYM2 does. MGA is also potentially related to ATF7IP-mediated silencing of germ cell-related genes. It forms a complex with MAX [26–28] and plays a role in the silencing of germ cell-related genes in mESCs [26, 29]. We observed that the dFNIII mutant-rescued cells still showed increased expression of some germ cell-related genes (Fig. 5d). This observation, together with the function of MGA, implies the possibility of the involvement of MGA in ATF7IP-mediated silencing via interaction with the FNIII domain.

## Conclusions

**Fig. 6:**
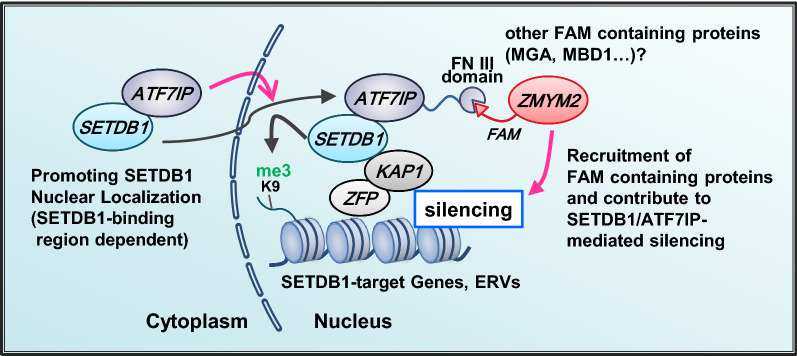
Proposed model for the role of SETDB1-binding region and FNIII domain of ATF7IP in the SETDB1/ATF7IP complex-mediated silencing

We revealed that the SETDB1-interacting region of ATF7IP is essential for its transcriptional silencing function, but the FNIII domain is mostly dispensable for the silencing of SETDB1/ATF7IP-targeted genes and retroelements. Only some germ cell-related genes expressed in *Atf7ip* KO ESCs were not silenced by the FNIII domain mutant. The silencing potential of the FNIII domain mutant against SETDB1-targeted retroelements is weaker than that of WT ATF7IP, suggesting that the FNIII domain plays a role as one of the multiple layers in the SETDB1/ATF7IP complex-mediated transcriptional silencing (Fig. 6). We also identified binding proteins for the FNIII domain and their consensus-binding motif. In addition, we showed that one of the FNIII domain-binding proteins, ZMYM2, partly mediates the efficient silencing of the transgene by ATF7IP. Taken together, this study contributes to the understanding of the function of ATF7IP and its domains.

## Methods

### Cell culture and DNA transfection

mESCs were cultured in Dulbecco’s modified Eagle’s medium (DMEM, Cat# D6429; Sigma-Aldrich, USA) supplemented with 15% KnockOut Serum Replacement (KSR; Cat# 10828028, Invitrogen, USA), 1% fetal bovine serum, 0.1 mM β-mercaptoethanol, leukemia-inhibiting factor, and 1× nonessential amino acids. HEK293T cells were cultured in DMEM (Cat# 08457-55; Nacalai Tesque, Japan) supplemented with 10% FBS. The mESC lines used in this study as follows: *Atf7ip* KO (clone name: TT#2-12), *Zmym2* KO (clone name: #1 and #2), and *Atf7ip*/*Zmym2* DKO (clone name: #1 and #2). *Zmym2* KO and *Zmym2*/*Atf7ip* DKO cells were established by CRISPR/Cas9 technology using pKLV2-U6gRNA5(Bbs1)-PGKpuro2ABFP [30]. Target sequences of gRNAs are listed in Additional file 12: Table S3. Stably transfected cell lines were established by the piggyback transposon system. DNA transfection was performed using Lipofectamine 2000 (Invitrogen, USA; for mESCs) or Polyethylenimine Max (Polyscience, Inc., USA; for HEK293T cells), according to the manufacturer’s instructions.

### Plasmids

Full-length mouse ATF7IP expression vector was described previously [22]. The deletion mutants of ATF7IP was generated by PCR-based method. The FNIII domain (residues 1190–1306) cDNA was inserted into pColdGST vector (Takara Bio Ltd., Japan). Full-length mouse *Zmym2* cDNA was generated from FANTOM clones AK135499 and AK137720. The *Zmym2* cDNA was inserted into pPB-CAG-V5-IRES.puromycine vector by In-Fusion technology. Full-length mouse *Mbd1* cDNA was PCR-amplified from FAMTOM clone AK046252 and inserted into pPB-CAG-V5-IRES.puromycine vector by In-Fusion technology. Full-length mouse *Zfp518a* cDNA was PCR-amplified from cDNA of mESCs and inserted into pPB-CAG-3xFLAG-IRES.puromycine vector by In-Fusion technology. *Mga* 2362–3003, mouse *Kiaa1551,* and mouse *Zmym4* with its 5′UTR cDNAs were PCR-amplified from total cDNA of mESCs and inserted into pPB-CAG-V5-IRES.puromycine vector by In-Fusion technology. All the mutant vectors were produced by overlapping PCR with primers harboring mutations. The detailed information of plasmids and the used primers were given in supplemental file (Additional file 9: Table S2).

### Purification of recombinant proteins

*Escherichia coli* (*E. coli*) BL21 (pLysS) strains were transformed with expression vectors, and the transformed bacteria were cultured in 2× YT medium with antibiotics and 0.5 mM isopropyl β-d-1-thiogalactopyranoside (IPTG) for 16 h at 16 °C. For purification of GST-tagged proteins, the cultured cell pellets were lysed with lysis buffer (1xPBS/0.5% NP-40) added with phenylmethanesulfonyl fluoride (PMSF) at 1 mM. After sonication with a Branson Sonifier (S-250D, Branson Ultrasonics) for 10 or 20 min on ice, the lysates were centrifuged at 7300×*g* for 10 min. The supernatants were incubated with prewashed Glutathione 4B sepharose (GE) for 1–3 h at 4 °C with gentle rotation. The beads were washed five times with the lysis buffer and then eluted with elution buffer (50 mM Tris, 10 mM Glutathione, 1 mM dithiothreitol (DTT)). The eluted proteins were dialyzed with 1xPBS/10% glycerol, and the concentration was measured by the Bradford protein assay and SDS–PAGE.

### Western blot and quantitative PCR analysis

Both analyses were performed as described previously [22]. The antibodies used in this study are described in “Antibodies” section.

The primers used in qPCR analysis are provided in Additional file 9: Table S2.

### Immunoprecipitation

For immunoprecipitation (IP), cells were lysed with normal-lysis buffer (50 mM Tris–HCl at pH 7.5, 150 mM NaCl, 1 mM EDTA, 10% glycerol, 1% NonidetP-40, 1 mM PMSF, 1× protease inhibitor cocktail), mid-lysis buffer (50 mM Tris–HCl at pH 7.5, 300 mM NaCl, 1 mM EDTA, 10% glycerol, 1% NonidetP-40, 1 mM phenylmethanesulfonyl fluoride (PMSF), 1× protease inhibitor cocktail; Nacalai Tesque, Japan) or the high-salt lysis buffer (50 mM Tris–HCl at pH 7.5, 500 mM NaCl, 1 mM EDTA, 10% glycerol, 1% Nonidet P-40, 1 mM PMSF, 1× protease inhibitor cocktail; Nacalai Tesque, Japan). After centrifugation at 14,000×*g* for 10 min, the supernatants were incubated with anti-FLAG affinity gel (Sigma-Aldrich, USA) or antibody-conjugated Protein A and Protein G Dynabeads mix for at least 3 h to overnight at 4 °C. The resin was then washed five times with the lysis buffer and eluted by 2× Laemmli sample buffer. Equivalent amounts of the input and the precipitates were subjected to standard western blot analysis.

### Antibodies

Following antibodies used for this study: anti-α-Tubulin (clone B-5-1-2, Sigma-Aldrich); anti-FLAG M2 antibody (F3165, Sigma-Aldrich for western blot and IF assays; F7425, Sigma-Aldrich for IF analysis); anti-MCAF1/ATF7IP (ab84497, Abcam); anti-SETDB1/ESET (CP10377, CELL APPLICATIONS, for western blot and IF analyses); anti-V5 (R960-25, Thermo Fisher which is the same as #46-0705, Life technology); anti-ZMYM2 (A301-711A, Bethyl Laboratories).

### Mass spectrometry analysis for identification of the ATF7IP FNIII domain-binding proteins

mESCs cultured in two 15-cm dishes (approximately 6.0 × 10^7^ cells/dish) were collected, lysed with 8 mL Buffer A (10 mM HEPES at pH 7.9, 10 mM KCl, 1.5 mM MgCl_2_, 0.34 M sucrose, 10% glycerol, 1 mM DTT, 1 mM PMSF, 1× protease inhibitor cocktail) containing Triton-X 100 at 0.1% as a final concentration and incubated on ice for 5 min. After centrifugation at 1300×*g* for 4 min at 4 °C, the pellet (nuclear fraction) was re-suspended in 2 mL normal-lysis buffer and was sonicated. After centrifugation at 14,000×*g* for 10 min, the supernatants were incubated with 100 µL of Glutathione 4B sepharose (50% slurry) for 1 h. The supernatant was then incubated with the recombinant FNIII domain-immobilized Glutathione 4B sepharose (2 µg protein/10 µL beads volume) at 4 °C overnight. The resin was then washed seven times with the lysis buffer and washed two times additionally with 100 mM ammonium bicarbonate (ABC) buffer. The pellet was re-suspended in 100 µL ABC buffer with 1/10 volume of acetonitrile and DTT (20 mM). The mixture was incubated at 56 °C for 30 min and then 6 µL of 500 mM iodoacetoamide was added and incubated at 37 °C for 30 min in the dark. The proteins were digested with 3 µg Tripsin/Lys-C mix (Promega) at 37 °C overnight. The digested protein fragments were applied to quantitative MS/MS analysis and following protein identification as described previously [31].

### Immunostaining

Immunostaining was performed as described previously with some minor modifications [32]. mESCs (8.0 × 10^4^) were seeded on 8-well Chamber (192-008, WATSON) which was precoated with 10 μg/mL of laminin for at least 2 h at 37 °C. After overnight culture, the cells were washed with PBS twice and fixed with 4% paraformaldehyde for 20 min at room temperature (RT). After fixation, the cells were permeabilized with 0.2% Triton X-100 in PBS for 10 min at RT and were then incubated with 3% BSA/0.2% Tween-20 in 4xSSC for 30 min at RT and with primary antibody for additionally 2 h at RT. After washing twice with 4xSSC, the cells were incubated with secondary antibodies conjugated with Alexa Fluor for 1 h at RT, washed with 4xSSC twice, and finally mounted with ProLong Diamond Antifade Mountant with DAPI (P36961, Thermo Fisher Scientific). Images were obtained using a confocal microscope (FV3000, Olimpus, Japan) and analyzed by Image J (1.50i). The obtained data were further analyzed by R (3.4.1).

### ChIP assay

For crosslinked ChIP (X-ChIP) analysis, 1 × 10^7^ cells were suspended in 1 mL PBS containing 10% FBS, and 62.5 µL of 16% formaldehyde (methanol free) was added to the cell suspension. After 10 min incubation at 25 °C, the cross-linking was quenched by the addition of 100 µL of 2.5 M glycine. After centrifugation at 6500×*g* for 3 min at 4 °C, the pellets were briefly washed with ChIP-lysis buffer (10 mM Tris–HCl, pH 7.5, 10 mM NaCl, 0.5% NP-40, and 1× protease inhibitor cocktail) and were then incubated with 500 µL of the ChIP-lysis buffer for 5 min on ice. After centrifugation at 6500×*g* for 3 min at 4 °C, the pellets were re-suspended in SDS-lysis buffer (50 mM Tris–HCl, pH 8.0, 10 mM EDTA, 1% SDS, and 1× protease inhibitor cocktail). After 10 min incubation on ice, 400 µL of Triton buffer (15 mM Tris–HCl, pH 8.0, 150 mM NaCl, 1 mM EDTA, 1% Triton X-100, and 1× protease inhibitor cocktail) was added to the suspensions. The cell lysates were then sonicated using a Bioruptor UCD-250 (Diagenode). After centrifugation at 14,000×*g* for 10 min at 4 °C, the supernatants were diluted with Triton buffer to 1100 µ. We used 100 µL or 1000 µL of the lysates for input samples or IP samples, respectively. For IP, the lysates were incubated with antibody-conjugated magnetic beads (Dynabeads M-280 Sheep antimouse IgG or Dynabeads Protein G, Invitrogen) at 4 °C for overnight. After sequential washes with low-salt buffer (20 mM Tris–HCl, pH 7.5, 150 mM NaCl, 2 mM EDTA, 1% Triton X-100, 0.1% SDS), high-salt buffer (20 mM Tris–HCl, pH 7.5, 500 mM NaCl, 2 mM EDTA, 1% Triton X-100, 0.1% SDS), LiCl buffer (10 mM Tris–HCl, pH 7.5, 25 mM LiCl, 1 mM EDTA, 1% Deoxycholate, 1% NP-40) and TE, the bound DNA was recovered and then analyzed by qPCR.

### RNA-seq analysis

The total RNA was prepared as described above. Sequencing libraries for transcriptome analysis were prepared using TruSeq Stranded mRNA LT Sample Prep Kit (Illumina) and sequenced using HiSeq 2500 (Illumina Inc.). Raw FastQ data were trimmed with Trim Galore (v0.3.7, default parameters) (http://www.bioinformatics.babraham.ac.uk/projects/trim_galore/) and mapped to the mouse GRCm38 genome assembly and UCSC genes database from the UCSC genome browser using TopHat (v2.1.1) [33]. After read mapping, mapped reads were analyzed by TEtranscripts (v1.4.11, default parameters) [34] to calculate gene and repeat expression levels and identify DE genes and repeats (adj. *P* value < 0.05, FC > 2). UCSC genes database and RepeatMasker track from the UCSC genome browser were used for the calculation of gene and repeat expression levels, respectively.

### ChIP-seq analysis

ChIP-seq libraries were prepared using KAPA Hyper Prep Kit (KAPA Biosystems) and sequenced using HiSeq 2500 (Illumina Inc.). The Raw FastQ data were processed as described above. Processed reads were mapped to the mouse GRCm38 genome assembly using Bowtie (v4.4.6) [35]. Peaks were identified by MACS (v1.4.1) [36]. UCSC genes database and RepeatMasker track from the UCSC genome browser were used for gene and repeat annotations, respectively. Enrichment of ChIP-seq data on retroelement was analyzed by ngsplot v2.47.1 [37].

## Supplementary information

**Additional file 1: Fig. S1.** Related to Fig. 1. **A**, **B** Sequence alignment of human and mouse ATF7IP for SETDB1-binding region (A) or FNIII domain (B).

**Additional file 2: Fig. S2.** Related to Fig. 2. **A** IF analysis shows that exogenous expression of 3xFLAG-ATF7IP WT and dFNIII mutant rescues the abnormal localization of SETDB1 in *Atf7ip* KO mESCs at day 5. Representative images are shown, and the quantitative analyses are shown in B and C. Scale bar: 10 µm. **B**, **C** SETDB1 (B) and 3xFLAG-ATF7IP (C) signals in the nucleus that was determined by DAPI staining were calculated. The mean from three independent experiments is shown as a bar graph with jittered points indicating the average % intensity of each experiment. Over 100 cells were analyzed from a single experiment.

**Additional file 3: Table S1.** Related to Fig. 3. List of FNIII domain-binding proteins. Proteins highlighted in light blue, light orange, light yellow, and light green are the validated proteins, the LCH (LSD1-CoREST-HDAC1) complex, the bait (ATF7IP) and the CPSF (cleavage and polyadenylation specificity factor) complex.

**Additional file 4: Fig. S3.** Related to Fig. 3. STRING analysis (https://string-db.org/) reveals several protein–protein interaction networks in the identified top 20 proteins. LCH complex components: Kdm1a, Rcor2, Hdac1 and Hdac2, CPSF complex components: Cpsf2 and Cstf2t.

**Additional file 5: Fig. S4.** Related to Fig. 3. **A** Schematic of ZMYM2 protein and a series of its mutants. The residues 182–350 was predicted as the region important for the interaction with ATF7IP. Mym domains were shown to interact with LSD1-HDAC complex. **B**–**D** Co-IP experiments using each ZMYM2 mutants.

**Additional file 6: Fig. S5.** Related to Fig. 3. **A** Co-IP experiment in HEK293T cells shows that mutations at the FAM impede the interaction of MGA C-terminus with ATF7IP. **B** Co-IP experiment in HEK293T cells shows that mutations at or deletion of the FAM impede the interaction of ZFP518A with ATF7IP. **C** Co-IP experiment in HEK293T cells shows that mutations at the FAM impede the interaction of ZMYM4 with ATF7IP.

**Additional file 7: Fig. S6.** Related to Fig. 4. **A** The transfection of *Zmym2* gRNA-expressing vector resulted in a slight increase in the expression of MSCV-GFP reporter, as evidenced by flow cytometric analysis. The *Atf7ip* gRNA-expressing vector was used as a positive control. **B** The established *Zmym2* KO cell lines show no increase in the expression of MSCV-GFP reporter, as evidenced by flow cytometric analysis. *Atf7ip* KO mESCs were used as a positive control. **C** Re-expression of 3xFLAG-ZMYM2 in *Zmym2* KO mESCs repressed the genes upregulated commonly in *Atf7ip* KO mESCs expressing the dFNIII mutant and the *Zmym2* KO mESCs (*Fkbp6, MaeI* and *Rec114*) or specific in *Zmym2* KO mESCs (*1700019A02Rik*). The data is representative of reproducible results of multiple experiments. Data are mean ± SEM; NS: P > 0.05, *P < 0.05, **P < 0.001 by Tukey’s test.

**Additional file 8: Fig. S7.** Related to Fig. 5. **A** The number of replicates of RNA-seq and ChIP-seq.** B** Correlation between replicates. Scatter plot of log2 (RPM + 1) of genes between replicate. The figure was generated using the smoothScatter function in R software version 3.5.1.** C** PCA of RNA-seq data. Principal component analysis (PCA) of gene expression among RNA-seq libraries. The first two components, PC1 and PC2, define the x- and y-axes of the two-dimensional space, respectively. The distance between two points reflects the variance in gene expression between them. PC1 and PC2 accounted for 35.9% and 16.93%, respectively, of the contribution to the variance. PCA was calculated using the prcomp function in R software version 3.5.1.

**Additional file 9: Table S2.** Related to Fig. 5. List of DE genes and repeats in *Atf7ip* KO, *Atf7ip* KO rescued with WT or the FNIII domain mutant of ATF7IP and *Zmym2* KO ESCs.

**Additional file 10: Fig. S8.** Related to Fig. 5. **A** Overlap of upregulated genes in *Atf7ip* KO and *Zmym2* KO ESCs. **B** Genes related to gametogenesis or meiosis are commonly derepressed in *Atf7ip* or *Zmym2* KO ESCs. **C** ATF7IP mainly represses ERV and Zmym2 mainly represses L1. **D** All retroelement except for IAPEy-int upregulated in *Atf7ip* KO mESCs are repressed by FN3 independent manner.

**Additional file 11: Fig. S9.** Related to Fig. 5. **A** Overlap of FLAG-ATF7IP WT peaks (replicate 1 + 2) and FNIII domain mutant stringent peaks. **B** IGV screenshots of RNA-seq and FLAG-ATF7IP ChIP-seq peaks of representative ATF7IP target genes (*Dazl* and *Fkbp6*).

**Additional file 12: Table S3.** Primers and plasmids.

## Data Availability

The ChIP-seq and RNA-seq data have been deposited in the National Center for Biotechnology Information Sequence Read Archive under accession numbers PRJNA664286. Other datasets used and analyzed during the current study are available from the corresponding author on reasonable request.
