# Supplementary material for: The fibronectin type-III (FNIII) domain of ATF7IP contributes to efficient transcriptional silencing mediated by the SETDB1 complex
Source: Epigenetics Chromatin. 2020 Nov 30;13:52. doi: 10.1186/s13072-020-00374-4 (PMC7706265; doi:10.1186/s13072-020-00374-4)
Supplement: Supplementary file 1 — Additional file 1: Fig. S1. Related to Fig. 1. A, B Sequence alignment of human and mouse ATF7IP for SETDB1-binding region (A) or FNIII domain (B). [file 13072_2020_374_MOESM1_ESM.pptx]

## Slide 1
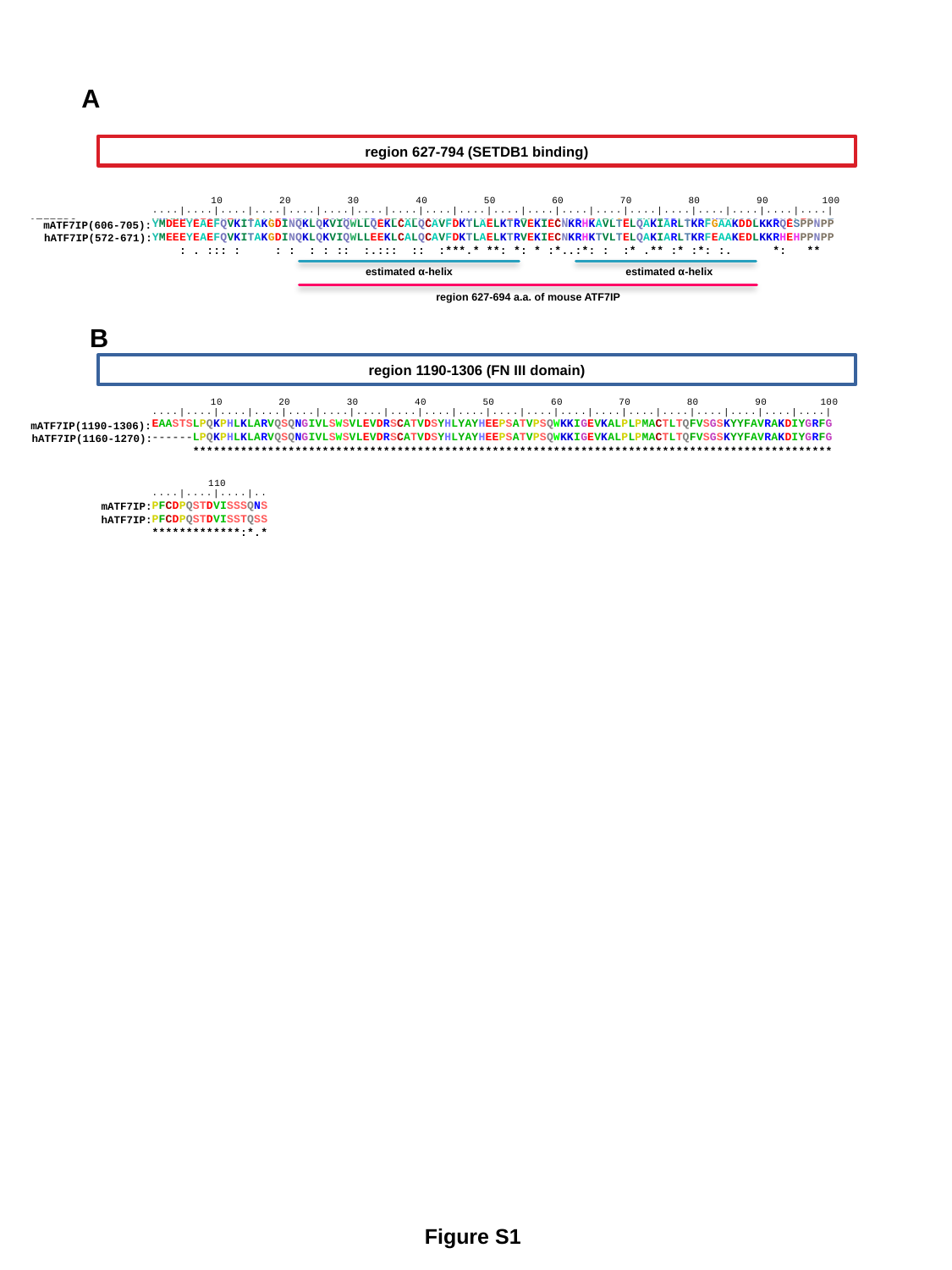

A
region 627-794 (SETDB1 binding)
mATF7IP(606-705):
hATF7IP(572-671):
estimated α-helix
estimated α-helix
region 627-694 a.a. of mouse ATF7IP
B
region 1190-1306 (FN III domain)
mATF7IP(1190-1306):
hATF7IP(1160-1270):
mATF7IP:
hATF7IP:
Figure S1
