# Supplementary material for: The fibronectin type-III (FNIII) domain of ATF7IP contributes to efficient transcriptional silencing mediated by the SETDB1 complex
Source: Epigenetics Chromatin. 2020 Nov 30;13:52. doi: 10.1186/s13072-020-00374-4 (PMC7706265; doi:10.1186/s13072-020-00374-4)
Supplement: Supplementary file 2 — Additional file 2: Fig. S2. Related to Fig. 2. A IF analysis shows that exogenous expression of 3xFLAG-ATF7IP WT and dFNIII mutant rescues the abnormal localization of SETDB1 in Atf7ip KO mESCs at day 5. Representative images are shown, and the quantitative analyses are shown in B and C. Scale bar: 10 µm. B, C SETDB1 (B) and 3xFLAG-ATF7IP (C) signals in the nucleus that was determined by DAPI staining were calculated. The mean from three independent experiments is shown as a bar graph with jittered points indicating the average % intensity of each experiment. Over 100 cells were analyzed from a single experiment. [file 13072_2020_374_MOESM2_ESM.pptx]

## Slide 1
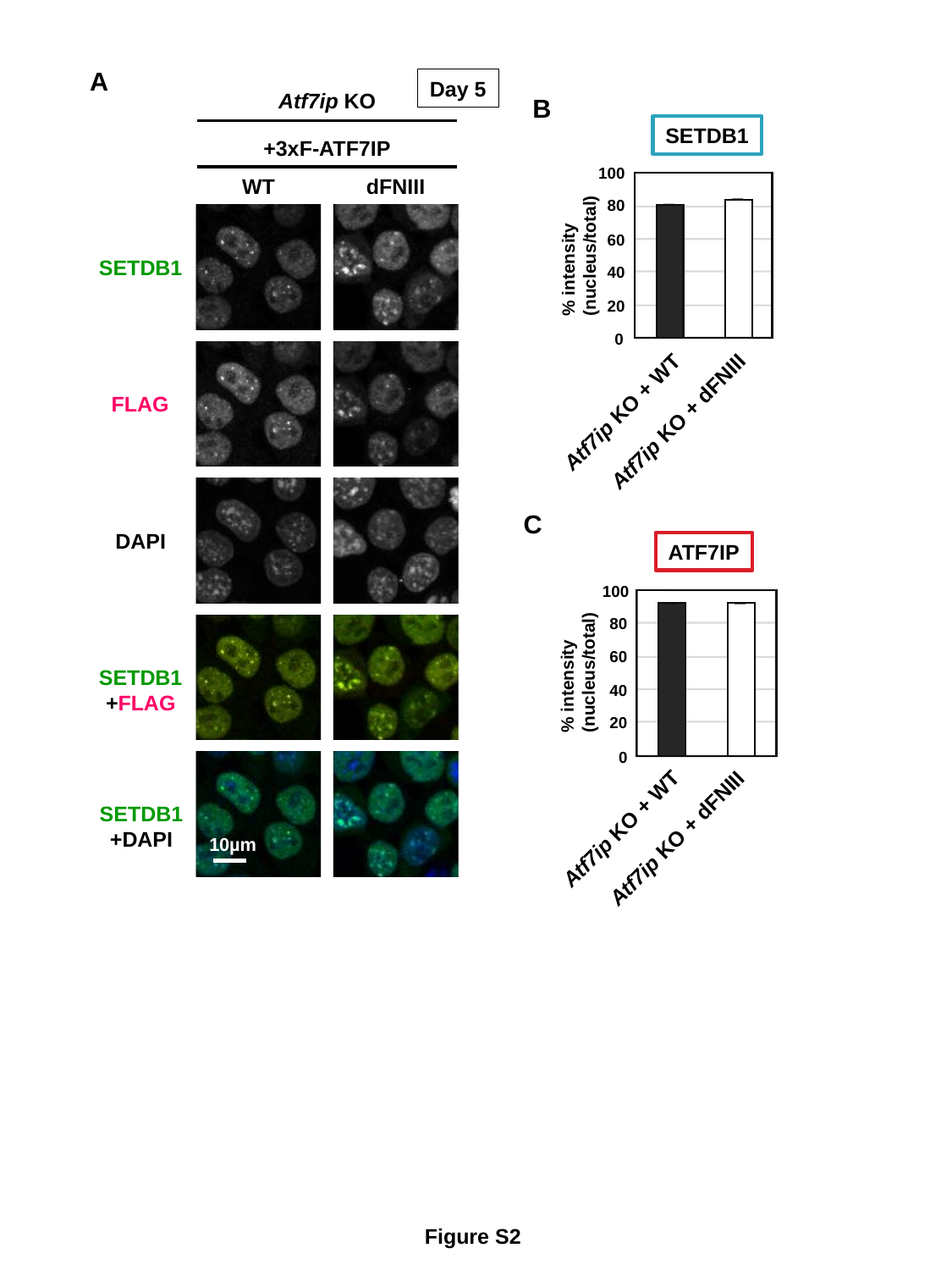

A
Day 5
Atf7ip KO
B
SETDB1
100
% intensity
(nucleus/total)
Atf7ip KO + WT
Atf7ip KO + dFNIII
80
60
40
20
0
+3xF-ATF7IP
WT
dFNIII
SETDB1
FLAG
C
DAPI
ATF7IP
100
% intensity
(nucleus/total)
Atf7ip KO + WT
Atf7ip KO + dFNIII
80
60
40
20
0
SETDB1
+FLAG
SETDB1
+DAPI
10µm
Figure S2
