# Supplementary figures and images for: The fibronectin type-III (FNIII) domain of ATF7IP contributes to efficient transcriptional silencing mediated by the SETDB1 complex
Source: Epigenetics Chromatin. 2020 Nov 30;13:52. doi: 10.1186/s13072-020-00374-4 (PMC7706265; doi:10.1186/s13072-020-00374-4)

## Slide 1
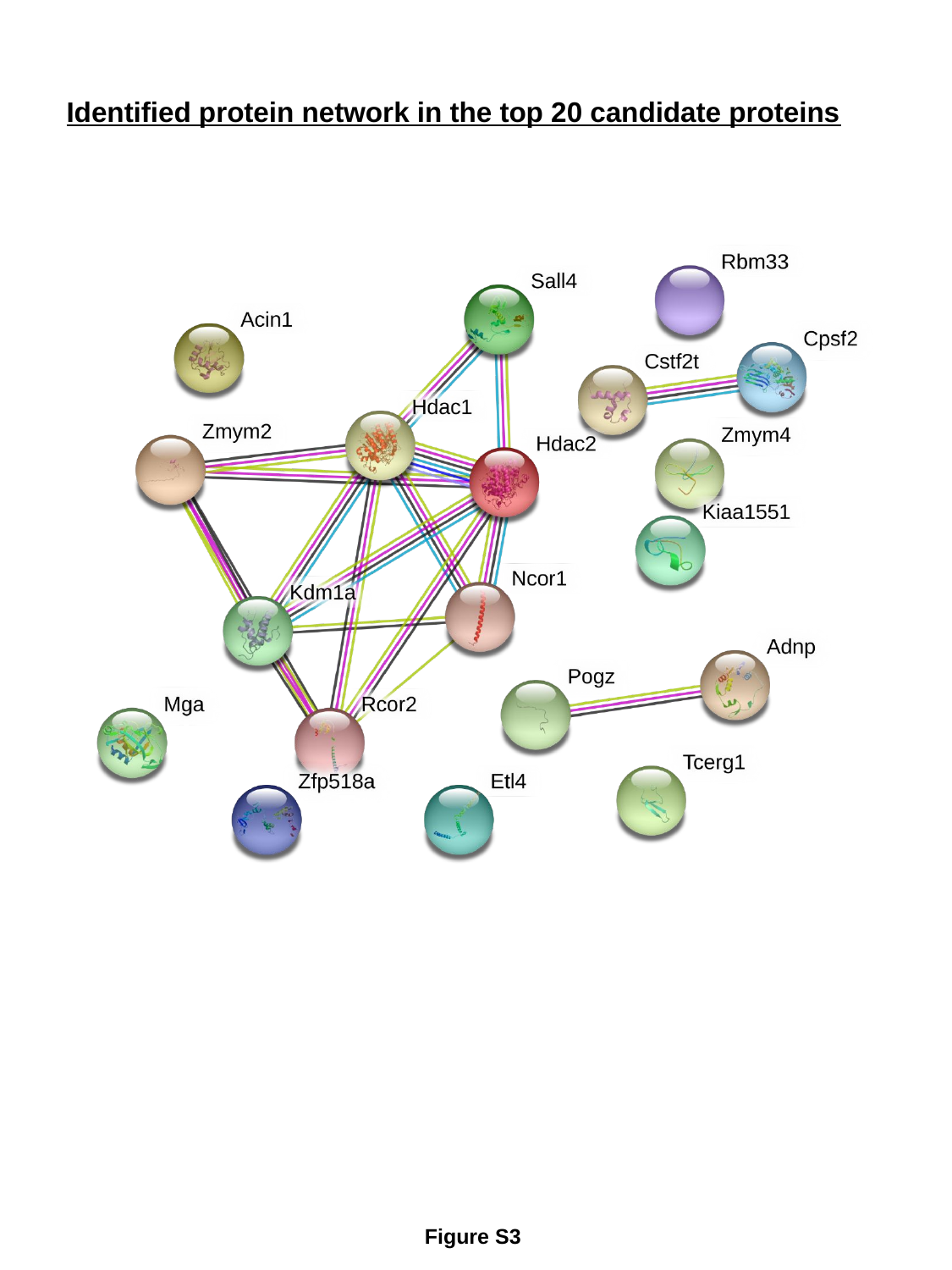

Identified protein network in the top 20 candidate proteins
Figure S3

Supplement: Supplementary file 4 — Additional file 4: Fig. S3. Related to Fig. 3. STRING analysis (https://string-db.org/) reveals several protein–protein interaction networks in the identified top 20 proteins. LCH complex components: Kdm1a, Rcor2, Hdac1 and Hdac2, CPSF complex components: Cpsf2 and Cstf2t. [file 13072_2020_374_MOESM4_ESM.pptx]

## Slide 1
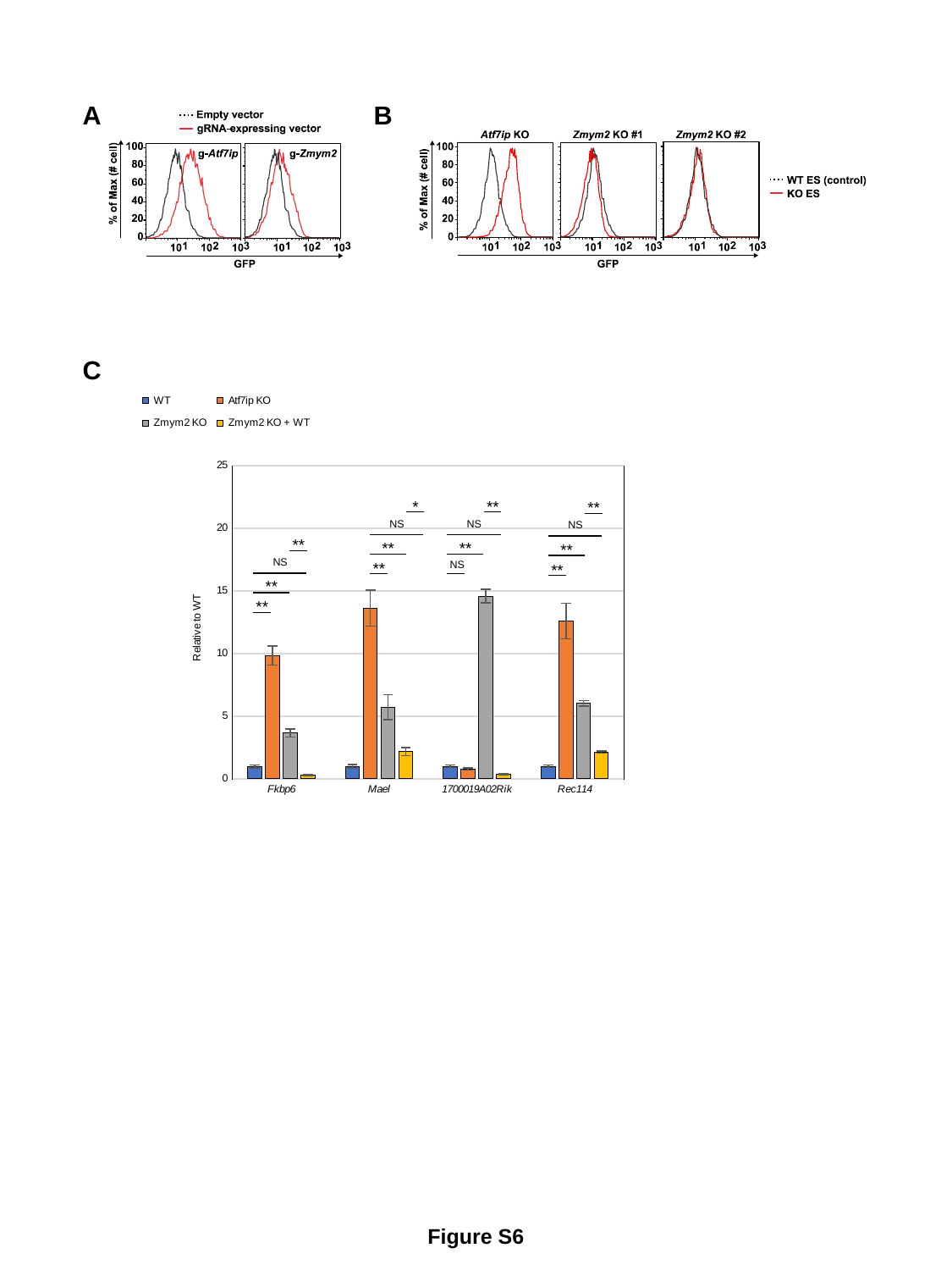

A
B
C
### Chart
| Category | WT | Atf7ip KO | Zmym2 KO | Zmym2 KO + WT |
|---|---|---|---|---|
| Fkbp6 | 1.0 | 9.852218862050815 | 3.66173484903808 | 0.27792278457074904 |
| Mael | 1.0 | 13.635466541297582 | 5.731148082065479 | 2.1674344473597413 |
| 1700019A02Rik | 1.0000000000000002 | 0.7870771629194149 | 14.588608499722858 | 0.3791171686837285 |
| Rec114 | 1.0 | 12.590409652692255 | 6.032284780276512 | 2.1369882052467397 |*
**
**
NS
**
**
NS
NS
**
**
**
NS
**
NS
**
**
Figure S6

Supplement: Supplementary file 7 — Additional file 7: Fig. S6. Related to Fig. 4. A The transfection of Zmym2 gRNA-expressing vector resulted in a slight increase in the expression of MSCV-GFP reporter, as evidenced by flow cytometric analysis. The Atf7ip gRNA-expressing vector was used as a positive control. B The established Zmym2 KO cell lines show no increase in the expression of MSCV-GFP reporter, as evidenced by flow cytometric analysis. Atf7ip KO mESCs were used as a positive control. C Re-expression of 3xFLAG-ZMYM2 in Zmym2 KO mESCs repressed the genes upregulated commonly in Atf7ip KO mESCs expressing the dFNIII mutant and the Zmym2 KO mESCs (Fkbp6, MaeI and Rec114) or specific in Zmym2 KO mESCs (1700019A02Rik). The data is representative of reproducible results of multiple experiments. Data are mean ± SEM; NS: P > 0.05, *P < 0.05, **P < 0.001 by Tukey’s test. [file 13072_2020_374_MOESM7_ESM.pptx]
