# Supplementary material for: The fibronectin type-III (FNIII) domain of ATF7IP contributes to efficient transcriptional silencing mediated by the SETDB1 complex
Source: Epigenetics Chromatin. 2020 Nov 30;13:52. doi: 10.1186/s13072-020-00374-4 (PMC7706265; doi:10.1186/s13072-020-00374-4)
Supplement: Supplementary file 5 — Additional file 5: Fig. S4. Related to Fig. 3. A Schematic of ZMYM2 protein and a series of its mutants. The residues 182–350 was predicted as the region important for the interaction with ATF7IP. Mym domains were shown to interact with LSD1-HDAC complex. B–D Co-IP experiments using each ZMYM2 mutants. [file 13072_2020_374_MOESM5_ESM.pptx]

## Slide 1
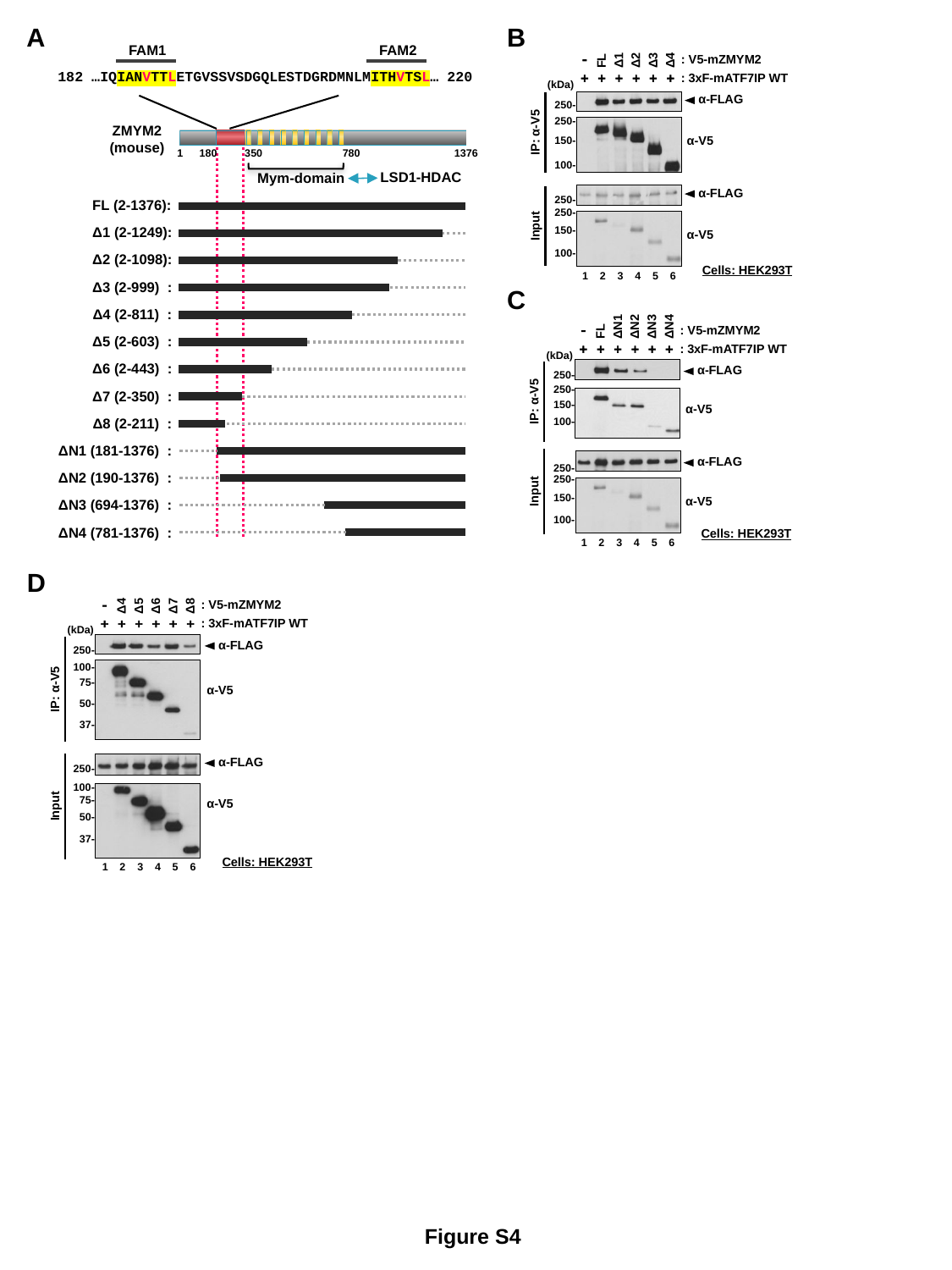

A
B
Δ2
Δ1
Δ4
FL
Δ3
-
FAM1
FAM2
182 …IQIANVTTLETGVSSVSDGQLESTDGRDMNLMITHVTSL… 220
: V5-mZMYM2
+
+
+
+
+
+
: 3xF-mATF7IP WT
(kDa)
α-FLAG
250-
250-
ZMYM2
(mouse)
1
180
350
780
1376
LSD1-HDAC
Mym-domain
FL (2-1376):
Δ1 (2-1249):
Δ2 (2-1098):
Δ3 (2-999) :
Δ4 (2-811) :
Δ5 (2-603) :
Δ6 (2-443) :
Δ7 (2-350) :
Δ8 (2-211) :
ΔN1 (181-1376) :
ΔN2 (190-1376) :
ΔN3 (694-1376) :
ΔN4 (781-1376) :
IP: α-V5
α-V5
150-
100-
α-FLAG
250-
250-
Input
150-
α-V5
100-
Cells: HEK293T
1
2
3
4
5
6
C
ΔN1
ΔN2
ΔN3
ΔN4
FL
-
: V5-mZMYM2
+
+
+
+
+
+
: 3xF-mATF7IP WT
(kDa)
α-FLAG
250-
250-
IP: α-V5
150-
α-V5
100-
α-FLAG
250-
250-
Input
150-
α-V5
100-
Cells: HEK293T
1
2
3
4
5
6
D
Δ6
Δ5
Δ8
Δ4
Δ7
-
: V5-mZMYM2
+
+
+
+
+
+
: 3xF-mATF7IP WT
(kDa)
α-FLAG
250-
100-
75-
IP: α-V5
α-V5
50-
37-
α-FLAG
250-
100-
75-
α-V5
Input
50-
37-
Cells: HEK293T
1
2
3
4
5
6
Figure S4
