# Supplementary material for: The fibronectin type-III (FNIII) domain of ATF7IP contributes to efficient transcriptional silencing mediated by the SETDB1 complex
Source: Epigenetics Chromatin. 2020 Nov 30;13:52. doi: 10.1186/s13072-020-00374-4 (PMC7706265; doi:10.1186/s13072-020-00374-4)
Supplement: Supplementary file 6 — Additional file 6: Fig. S5. Related to Fig. 3. A Co-IP experiment in HEK293T cells shows that mutations at the FAM impede the interaction of MGA C-terminus with ATF7IP. B Co-IP experiment in HEK293T cells shows that mutations at or deletion of the FAM impede the interaction of ZFP518A with ATF7IP. C Co-IP experiment in HEK293T cells shows that mutations at the FAM impede the interaction of ZMYM4 with ATF7IP. [file 13072_2020_374_MOESM6_ESM.pptx]

## Slide 1
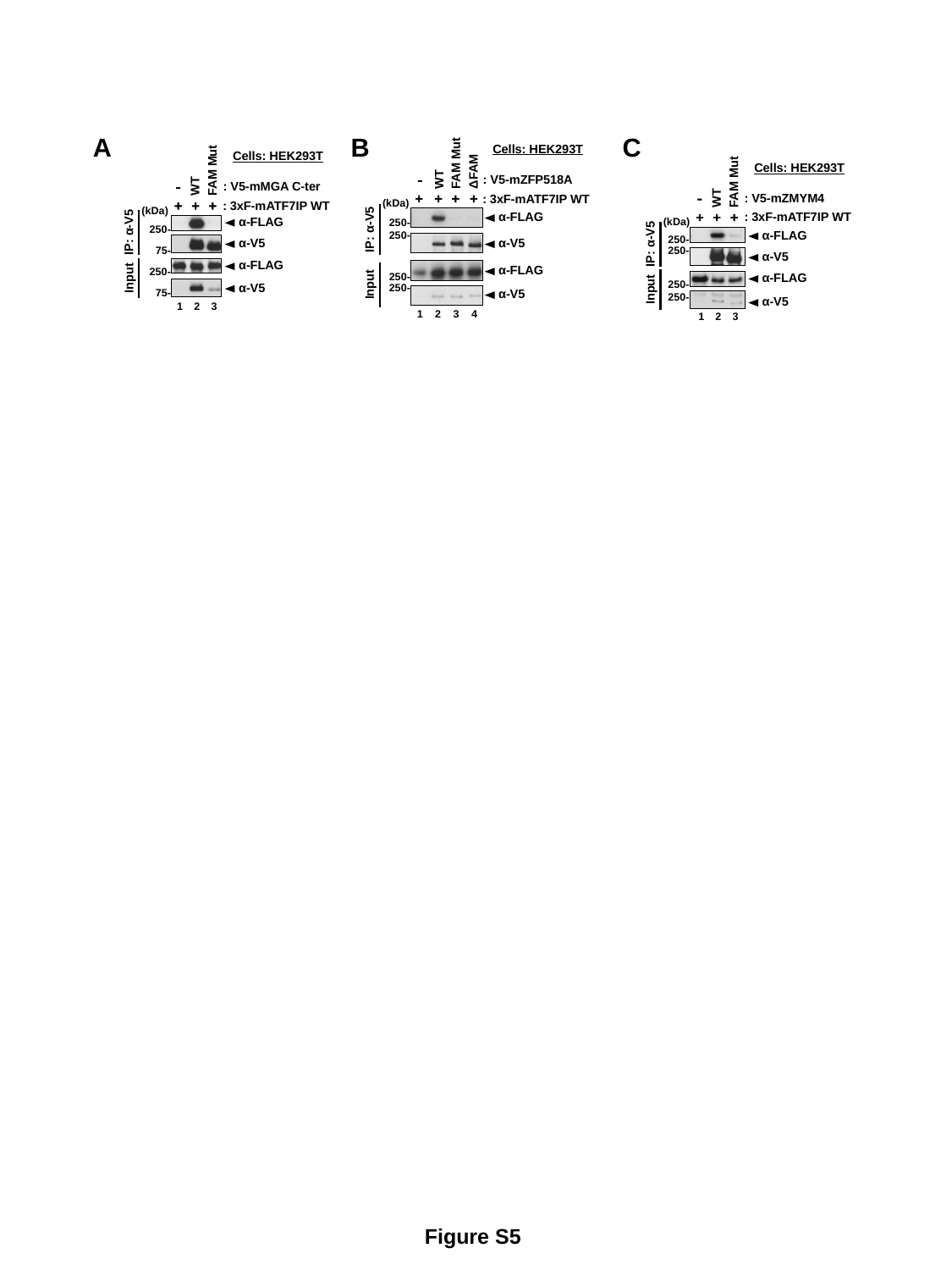

FAM Mut
Cells: HEK293T
WT
-
: V5-mMGA C-ter
+
+
+
: 3xF-mATF7IP WT
(kDa)
α-FLAG
250-
IP: α-V5
α-V5
75-
α-FLAG
250-
Input
α-V5
75-
1
2
3
FAM Mut
Cells: HEK293T
WT
-
: V5-mZMYM4
+
+
+
: 3xF-mATF7IP WT
(kDa)
α-FLAG
250-
IP: α-V5
250-
α-V5
α-FLAG
250-
Input
250-
α-V5
1
2
3
Cells: HEK293T
ΔFAM
FAM Mut
WT
-
: V5-mZFP518A
+
+
+
+
(kDa)
α-FLAG
250-
IP: α-V5
250-
α-V5
α-FLAG
250-
Input
250-
α-V5
1
2
3
4
: 3xF-mATF7IP WT
A
B
C
Figure S5
