# Supplementary material for: The fibronectin type-III (FNIII) domain of ATF7IP contributes to efficient transcriptional silencing mediated by the SETDB1 complex
Source: Epigenetics Chromatin. 2020 Nov 30;13:52. doi: 10.1186/s13072-020-00374-4 (PMC7706265; doi:10.1186/s13072-020-00374-4)
Supplement: Supplementary file 8 — Additional file 8: Fig. S7. Related to Fig. 5. A The number of replicates of RNA-seq and ChIP-seq. B Correlation between replicates. Scatter plot of log2 (RPM + 1) of genes between replicate. The figure was generated using the smoothScatter function in R software version 3.5.1. C PCA of RNA-seq data. Principal component analysis (PCA) of gene expression among RNA-seq libraries. The first two components, PC1 and PC2, define the x- and y-axes of the two-dimensional space, respectively. The distance between two points reflects the variance in gene expression between them. PC1 and PC2 accounted for 35.9% and 16.93%, respectively, of the contribution to the variance. PCA was calculated using the prcomp function in R software version 3.5.1. [file 13072_2020_374_MOESM8_ESM.pptx]

## Slide 1
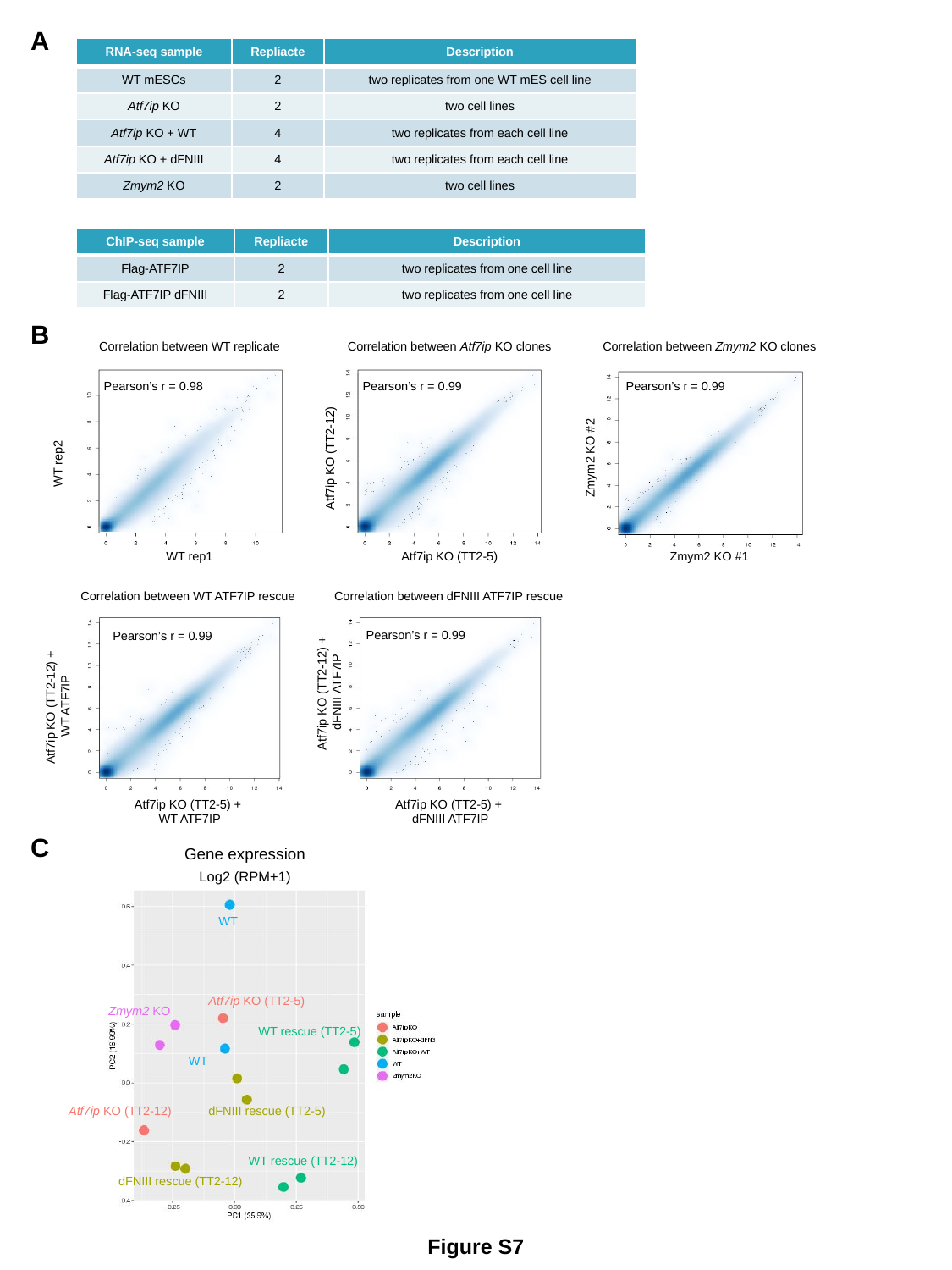

A
| RNA-seq sample | Repliacte | Description |
| --- | --- | --- |
| WT mESCs | 2 | two replicates from one WT mES cell line |
| Atf7ip KO | 2 | two cell lines |
| Atf7ip KO + WT | 4 | two replicates from each cell line |
| Atf7ip KO + dFNIII | 4 | two replicates from each cell line |
| Zmym2 KO | 2 | two cell lines |
| ChIP-seq sample | Repliacte | Description |
| --- | --- | --- |
| Flag-ATF7IP | 2 | two replicates from one cell line |
| Flag-ATF7IP dFNIII | 2 | two replicates from one cell line |
B
Correlation between WT replicate
Correlation between Atf7ip KO clones
Correlation between Zmym2 KO clones
Pearson’s r = 0.98
Pearson’s r = 0.99
Pearson’s r = 0.99
Atf7ip KO (TT2-12)
Zmym2 KO #2
WT rep2
WT rep1
Atf7ip KO (TT2-5)
Zmym2 KO #1
Correlation between WT ATF7IP rescue
Correlation between dFNIII ATF7IP rescue
Pearson’s r = 0.99
Pearson’s r = 0.99
Atf7ip KO (TT2-12) +
dFNIII ATF7IP
Atf7ip KO (TT2-12) +
WT ATF7IP
Atf7ip KO (TT2-5) +
WT ATF7IP
Atf7ip KO (TT2-5) +
dFNIII ATF7IP
C
Gene expression
Log2 (RPM+1)
WT
Atf7ip KO (TT2-5)
Zmym2 KO
WT rescue (TT2-5)
WT
Atf7ip KO (TT2-12)
dFNIII rescue (TT2-5)
WT rescue (TT2-12)
dFNIII rescue (TT2-12)
Figure S7
