# Supplementary material for: The fibronectin type-III (FNIII) domain of ATF7IP contributes to efficient transcriptional silencing mediated by the SETDB1 complex
Source: Epigenetics Chromatin. 2020 Nov 30;13:52. doi: 10.1186/s13072-020-00374-4 (PMC7706265; doi:10.1186/s13072-020-00374-4)
Supplement: Supplementary file 10 — Additional file 10: Fig. S8. Related to Fig. 5. A Overlap of upregulated genes in Atf7ip KO and Zmym2 KO ESCs. B Genes related to gametogenesis or meiosis are commonly derepressed in Atf7ip or Zmym2 KO ESCs. C ATF7IP mainly represses ERV and Zmym2 mainly represses L1. D All retroelement except for IAPEy-int upregulated in Atf7ip KO mESCs are repressed by FN3 independent manner. [file 13072_2020_374_MOESM10_ESM.pptx]

## Slide 1
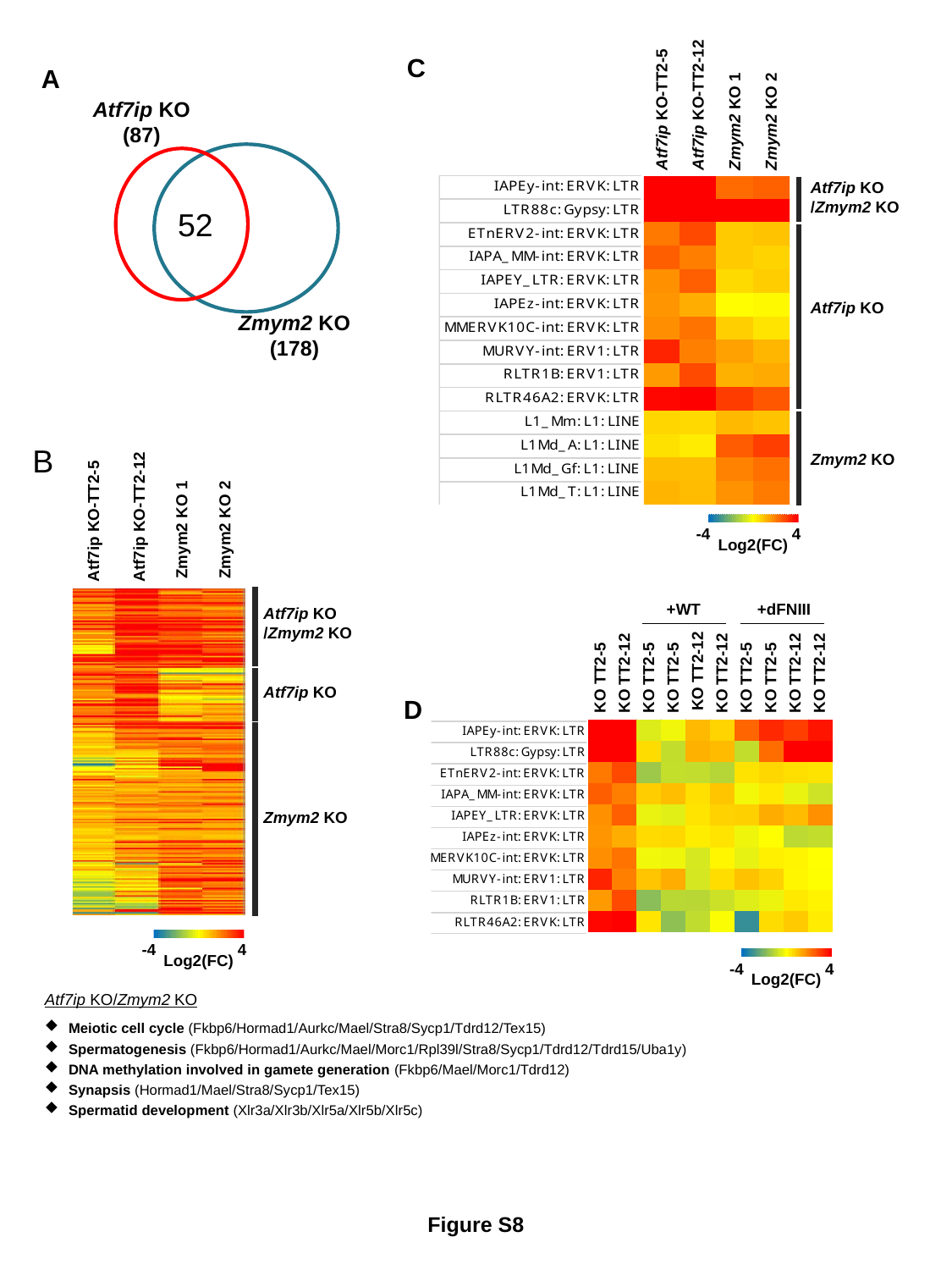

C
A
Atf7ip KO-TT2-5
Atf7ip KO-TT2-12
Zmym2 KO 1
Zmym2 KO 2
Atf7ip KO
(87)
52
Zmym2 KO
(178)
Atf7ip KO
/Zmym2 KO
Atf7ip KO
Zmym2 KO 1
Zmym2 KO 2
Atf7ip KO-TT2-5
Atf7ip KO-TT2-12
Atf7ip KO
/Zmym2 KO
Atf7ip KO
Zmym2 KO
B
Zmym2 KO
-4
4
Log2(FC)
+WT
+dFNIII
KO TT2-12
KO TT2-12
KO TT2-12
KO TT2-12
KO TT2-5
KO TT2-5
KO TT2-12
KO TT2-5
KO TT2-5
KO TT2-5
D
-4
4
Log2(FC)
-4
4
Log2(FC)
Atf7ip KO/Zmym2 KO
Meiotic cell cycle (Fkbp6/Hormad1/Aurkc/Mael/Stra8/Sycp1/Tdrd12/Tex15)
Spermatogenesis (Fkbp6/Hormad1/Aurkc/Mael/Morc1/Rpl39l/Stra8/Sycp1/Tdrd12/Tdrd15/Uba1y)
DNA methylation involved in gamete generation (Fkbp6/Mael/Morc1/Tdrd12)
Synapsis (Hormad1/Mael/Stra8/Sycp1/Tex15)
Spermatid development (Xlr3a/Xlr3b/Xlr5a/Xlr5b/Xlr5c)
Figure S8
