# Supplementary material for: The fibronectin type-III (FNIII) domain of ATF7IP contributes to efficient transcriptional silencing mediated by the SETDB1 complex
Source: Epigenetics Chromatin. 2020 Nov 30;13:52. doi: 10.1186/s13072-020-00374-4 (PMC7706265; doi:10.1186/s13072-020-00374-4)
Supplement: Supplementary file 11 — Additional file 11: Fig. S9. Related to Fig. 5. A Overlap of FLAG-ATF7IP WT peaks (replicate 1 + 2) and FNIII domain mutant stringent peaks. B IGV screenshots of RNA-seq and FLAG-ATF7IP ChIP-seq peaks of representative ATF7IP target genes (Dazl and Fkbp6). [file 13072_2020_374_MOESM11_ESM.pptx]

## Slide 1
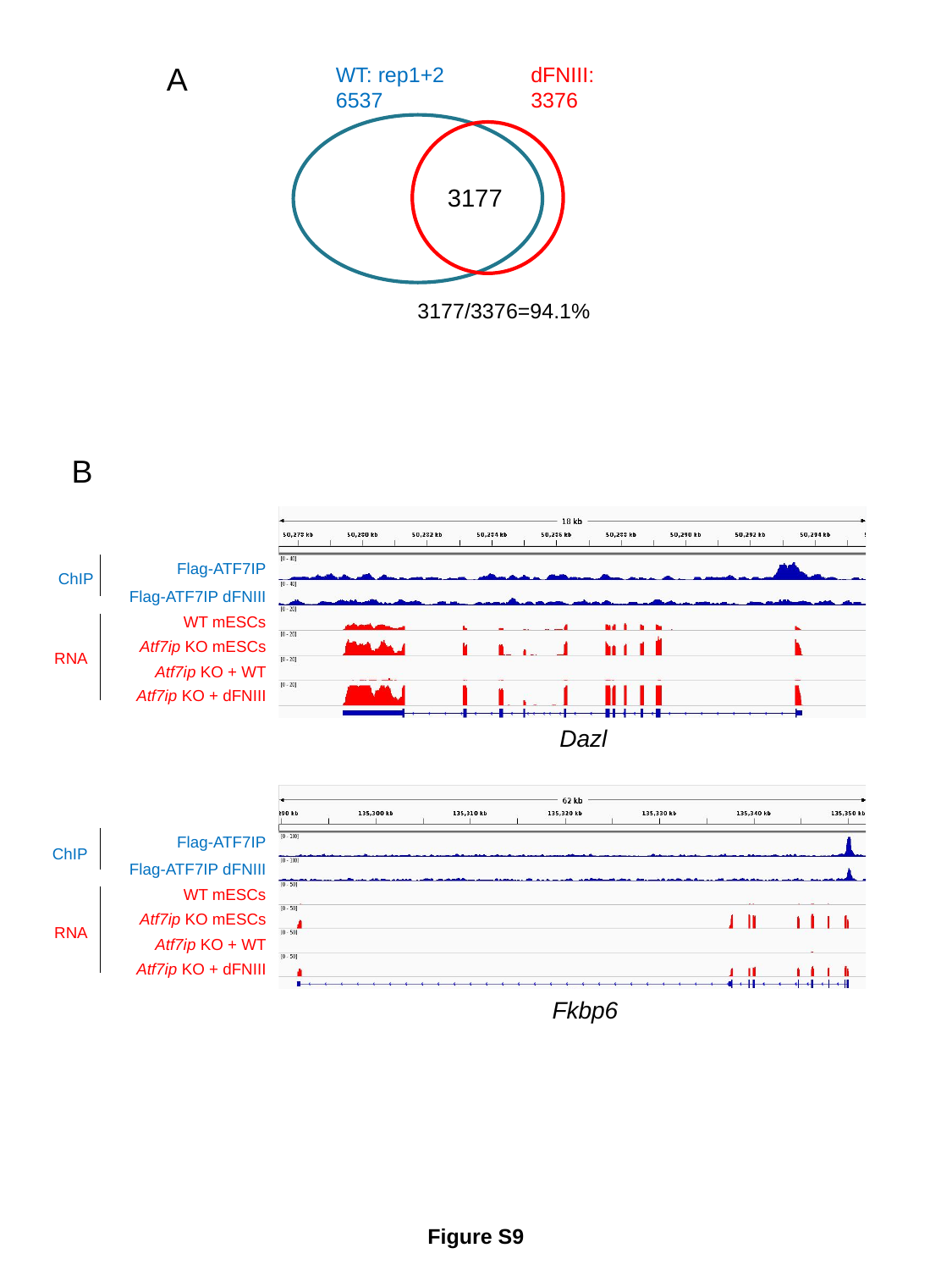

A
WT: rep1+2
6537
dFNIII:
3376
3177
3177/3376=94.1%
B
Flag-ATF7IP
ChIP
Flag-ATF7IP dFNIII
WT mESCs
Atf7ip KO mESCs
RNA
Atf7ip KO + WT
Atf7ip KO + dFNIII
Dazl
Flag-ATF7IP
ChIP
Flag-ATF7IP dFNIII
WT mESCs
Atf7ip KO mESCs
RNA
Atf7ip KO + WT
Atf7ip KO + dFNIII
Fkbp6
Figure S9
